# A Lucifer‐Based Environment‐Sensitive Fluorescent PNA Probe for Imaging Poly(A) RNAs

**DOI:** 10.1002/cbic.201700661

**Published:** 2018-03-13

**Authors:** Pramod M. Sabale, Uddhav B. Ambi, Seergazhi G. Srivatsan

**Affiliations:** ^1^ Department of Chemistry Indian Institute of Science Education and Research (IISER) Dr. Homi Bhabha Road Pune 411008 India

**Keywords:** fluorescent probes, imaging agents, peptide nucleic acids, poly(A) RNA, RNA

## Abstract

Fluorescence‐based oligonucleotide (ON) hybridization probes greatly aid the detection and profiling of RNA sequences in cells. However, certain limitations such as target accessibility and hybridization efficiency in cellular environments hamper their broad application because RNAs can form complex and stable structures. In this context, we have developed a robust hybridization probe suitable for imaging RNA in cells by combining the properties of 1) a new microenvironment‐sensitive fluorescent nucleobase analogue, obtained by attaching the Lucifer chromophore **(**1,8‐naphthalimide) at the 5‐position of uracil, and 2) a peptide nucleic acid (PNA) capable of forming stable hybrids with RNA. The fluorescence of the PNA base analogue labeled with the Lucifer chromophore, when incorporated into PNA oligomers and hybridized to complementary and mismatched ONs, is highly responsive to its neighboring base environment. Notably, the PNA base reports the presence of an adenine repeat in an RNA ON with reasonable enhancement in fluorescence. This feature of the emissive analogue enabled the construction of a poly(T) PNA probe for the efficient visualization of polyadenylated [poly(A)] RNAs in cells—poly(A) being an important motif that plays vital roles in the lifecycle of many types of RNA. Our results demonstrate that such responsive fluorescent nucleobase analogues, when judiciously placed in PNA oligomers, could generate useful hybridization probes to detect nucleic acid sequences in cells and also to image them.

## Introduction

Understanding endogenous RNA expression, processing, and transport is highly important for understanding of cell function and behavior in healthy and disease conditions. One of the important events, along with 5′‐capping, that controls the downstream processing of RNA, particularly mRNA, is 3′‐polyadenylation.^1^, ^2^ Most eukaryotic mRNAs and some viral mRNAs,^3^ contain a 3′‐poly(A) tail, formed by the addition of adenosine residues by poly(A) polymerases (PAPs).^4^ The 3′‐poly(A) tail in cooperation with the 5′‐cap structure influences mRNA splicing, promotes translation, and inhibits decay of mRNA.^4^, ^5^ Like mRNAs, many long noncoding RNAs and miRNAs are also processed from 5′‐capped and 3′‐polyadenylated transcripts.^6^ Studies indicate that changes in the length of the poly(A) tail and in the expression of PAP can affect mRNA stability, localization, and efficiency of translation.^7^ Notably, shorter isoforms resulting from alternative cleavage and polyadenylation of 3′‐untranslated regions of mRNAs have been implicated in the activation of oncogenes in a variety of human cancer cells.^8^ Hence, the poly(A) tail not only provides a way to access the functional state of mRNA but also serves as a potential marker for cancer. Consequently, there is considerable interest in developing robust methods for imaging and profiling polyadenylated RNAs.

Hybridization tools based on linear oligonucleotide (ON) probes and molecular beacons have greatly aided the visualization and profiling of cellular RNA.^9^ More recently, fluorescent aptamers^10^ and genetically encoded fusion proteins,^11^ as well as metabolic labeling of cellular RNA by use of nucleoside analogues, have also provided efficient systems to detect RNA in cells.^12^ In the case of poly(A) RNAs, hybridization assays based on bead‐modified poly(dT)/(U) ONs are used to isolate and study the polyadenylation state of cellular RNAs.^13^ Similarly, fluorescently modified oligo(dT) hybridization probes are used to visualize RNA sequences containing poly(A) tails in cells.^14^ Recently, metabolic labeling of cellular RNA with clickable adenosine analogues was used to monitor the polyadenylation process.^15^

Although these methods are highly useful, conventional ON hybridization probes suffer from certain shortcomings such as target accessibility, hybridization efficiency, and specificity, which impede their broader application when it comes to targeting cellular RNAs.9b This is because RNA sequences can adopt complex and stable secondary and tertiary structures, which are difficult to access and or invade with ON probes. To overcome the drawbacks of ON hybridization probes, several synthetic ON analogues have been developed by replacing or modifying the base or sugar‐phosphate backbone.^16^ In particular, peptide nucleic acids (PNAs), based on charge‐neutral pseudo‐peptide backbones [e.g., *N*‐(2‐aminoethyl)glycine (*aeg*)], offer unique advantages over conventional ON probes.^17^ Apart from being resistant to proteases and nucleases,^18^ PNAs bind more strongly to target nucleic acids than ONs, and efficiently invade duplex and other complex structures of nucleic acids.^19^ In addition, PNA**⋅**ON hybridization is significantly more affected than ON hybrids even by a single mismatched base pair and, hence, even short PNA oligomer probes offer significantly higher specificity than longer ONs used in in situ hybridization (ISH) assays.^20^ These features make PNAs very efficient oligomers in antisense and ISH applications for specific targeting and sensing of nucleic acids.^21^

The usefulness of PNAs as fluorescence in situ hybridization (FISH) probes in imaging different RNA sequences has been demonstrated with forced intercalation (FIT) PNA probes, in which one of the nucleobases is completely replaced with a fluorescent intercalator (e.g., thiazole orange).^22^ A FIT PNA probe shows low fluorescence, and upon binding to a complementary nucleic acid sequence it shows significant enhancement in fluorescence, thereby serving as a turn‐on sensor. As a demonstration of their utility, such probes have been elegantly implemented in hybridization assays to image various types of RNA sequences in cells in culture.^23^

**Figure 1:**
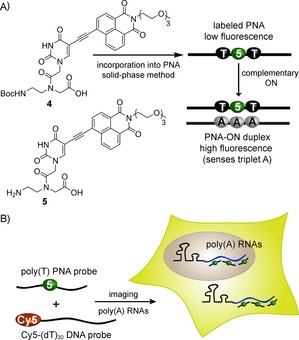
Imaging cellular poly(A) RNAs by using an environment‐sensitive fluorescent PNA base analogue based on the Lucifer (naphthalimide) chromophore. A) Chemical structures of naphthalimide‐modified uracil *aeg* PNA monomer **4** and its fully deprotected analogue **5**. PNA base analogue **5** reports the presence of a triplet adenine motif in an ON sequence through an enhancement in fluorescence intensity. B) A robust hybridization assay was designed by using a 12‐mer poly(T) PNA probe, incorporating base analogue **5**, to visualize poly(A) RNAs in cells. The short Lucifer‐labeled poly(T) PNA probe binds to poly(A) RNAs more effectively than Cy5‐dT_30_.

Alternatively, we hypothesized that a PNA oligomer labeled with an environment‐sensitive fluorescent PNA base analogue, which would retain its Watson–Crick hydrogen bonding ability and report a specific nucleobase environment in the PNA**⋅**ON duplex through a significant enhancement in fluorescence intensity, would provide new opportunities to visualize RNA sequences in cells effectively and with greater specificity. Herein, we describe the development of new microenvironment‐sensitive fluorescent PNA base analogue **5** (Figure 1), obtained by attaching the Lucifer chromophore (1,8‐naphthalimide) at the 5‐position of uracil. The fluorescence properties of **5** when incorporated into PNA**⋅**ON duplexes are highly responsive to the neighboring base environments. In particular, the PNA base analogue, when incorporated into a PNA oligomer and hybridized to complementary DNA and RNA ONs, reports the presence of adenine repeats in RNA through enhancement in fluorescence. This property of the emissive analogue, coupled with the inherent high stability and binding affinity of the PNA oligomer to the target RNA, has enabled the development of a robust method to visualize poly(A) RNAs in cells with the aid of a Lucifer‐labeled poly(T) PNA oligomer probe (Figure 1). Notably, this short 12‐mer PNA probe binds to poly(A) RNAs with significantly higher affinity than Cy5‐dT_30_, a commonly used DNA ON hybridization probe.

## Results and Discussion

### Design and synthesis of Lucifer‐modified PNA base analogue 5

A few environment‐sensitive PNA base analogues (e.g., 2‐aminopurine, 8‐vinylguanine, phenylpyrrolocytosine) have been incorporated into PNA oligomers and used as probes to study hybridization process and nucleic acid topologies.^24^ However, these base‐modified PNA oligomers show drastically quenched emission when hybridized to complementary ONs and/or exhibit emission profiles that are not compatible with conventional fluorescence microscopes. Our studies and literature reports indicated that responsive fluorescent nucleobase analogues can be generated by attaching heterocyclic moieties onto nucleobases.^25^ As part of our continuous efforts to develop responsive fluorescent probes, we identified the naphthalimide core of Lucifer dye as a promising heterocyclic moiety to build the fluorescent PNA building block for the following reasons.^26^ The Lucifer dye, based on a 1,8‐naphthalimide core, has an excitation and emission maximum in the visible region with a large Stokes shift and high quantum yield.^27^ The photophysical properties of the naphthalimide moiety, which mainly depend on the substituent present at the 4‐position, can be tuned by attaching different electron‐donating groups. Such rationally designed Lucifer derivatives are very useful as biological imaging markers.^28^ Therefore, we envisioned that conjugating a 4‐ethynyl‐1,8‐naphthalimide moiety at the 5‐position of uracil would generate a responsive *aeg* PNA base analogue with fluorescence properties suitable for nucleic acid analysis in both cell‐free and cellular environments (Figure 1).

**Scheme 1:**
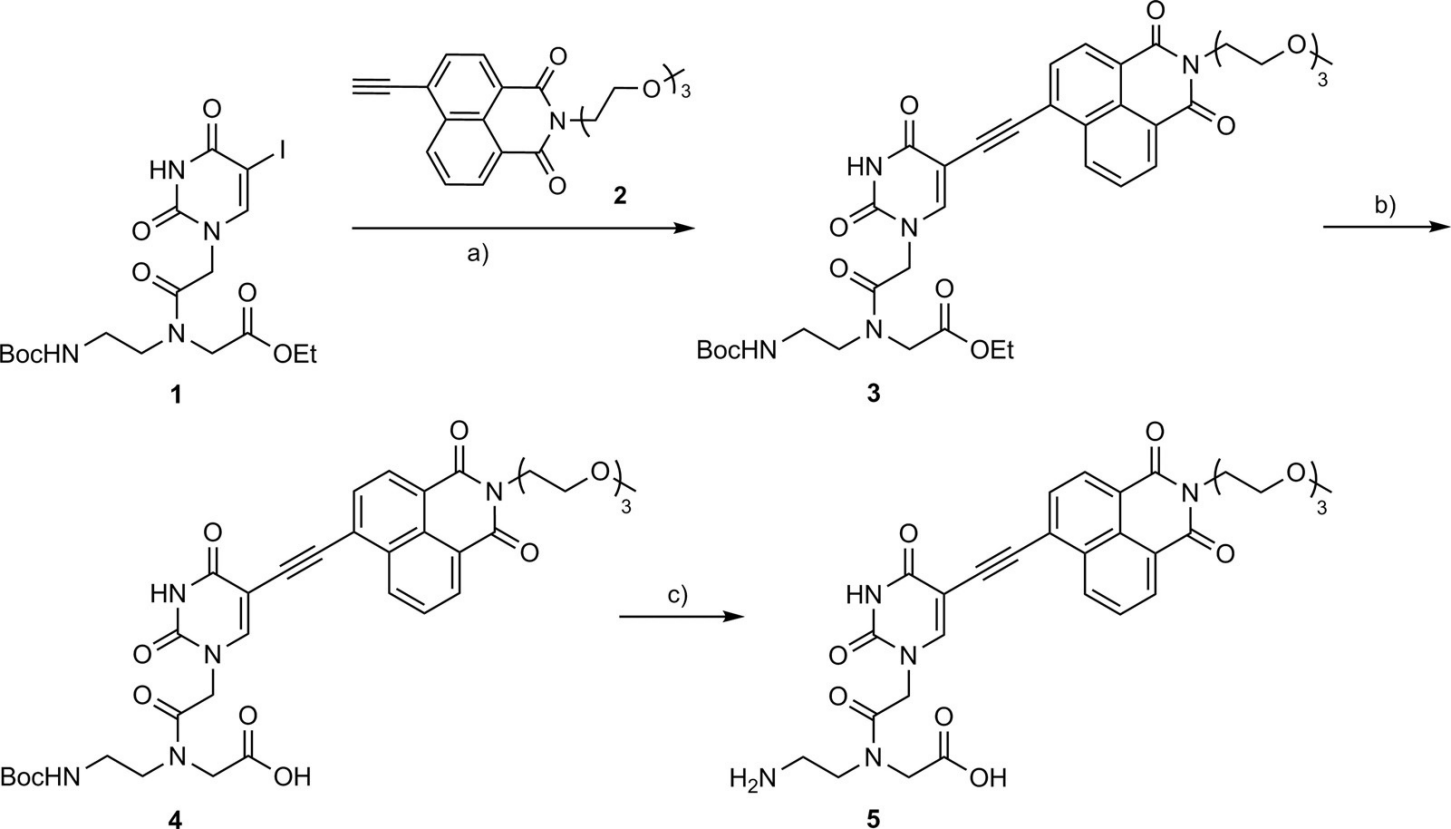
Synthesis of fluorescent naphthalimide‐modified uracil *aeg* PNA monomer **4** for SPPS. Fully deprotected analogue **5** was used in the photophysical analysis. a) Pd(PPh_3_)_4_, CuI, *i*Pr_2_NEt, THF, RT (40 %); b) LiOH, methanol, RT (69 %); c) 50 % TFA in CH_2_Cl_2_, RT (75 %).

The naphthalimide‐modified *aeg* PNA monomer **4** and its fully deprotected analogue **5**—required for solid‐phase PNA synthesis (SPPS) and photophysical characterization, respectively—were prepared by means of the steps illustrated in Scheme 1. 5‐Iodouracil *aeg* ethyl ester derivative **1** 29 and 4**‐**ethynyl**‐**1,8**‐**naphthalimide derivative **2** (containing a tri(ethylene glycol) group at the imide position, to improve solubility in aqueous media), synthesized in a separate set of reactions, were coupled under Sonogashira reaction conditions. Deprotection of the ester group afforded the naphthalimide‐modified PNA monomer **4** necessary for SPPS in reasonable yields. Further removal of the Boc group gave the PNA base **5** for photophysical analysis.

### Microenvironment‐sensitivity of Lucifer‐modified PNA base analogue 5

**Figure 2:**
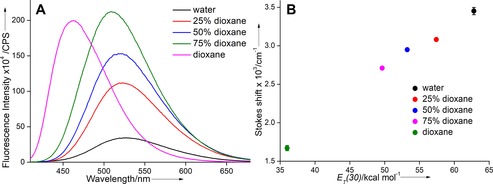
A) Emission spectra (5 μm) of PNA base analogue **5** in water, dioxane, and water/dioxane mixtures. Each sample was excited at its lowest energy maximum (Table 1). Excitation and emission slit widths were kept at 1 and 3 nm, respectively. All solutions contained 0.5 % DMSO. B) A plot of Stokes shift versus *E*_T_ *(30)* for **5** in solvents of different polarity.

UV absorption and fluorescence analyses of **5** were carried out in water, dioxane, and water/dioxane mixtures to evaluate the responsiveness of the PNA base analogue to changes in its microenvironment. Such a solvent combination is commonly used to study the effect of microscopic polarity on the emission properties of fluorescent probes.^30^ The absorption maximum in dioxane and water/dioxane mixtures was slightly red‐shifted relative to that in water, and a noticeable hyperchromic effect was observed in water/dioxane mixtures relative to in pure water and dioxane (Figure S1 A in the Supporting Information and Table 1). Interestingly, excited‐state properties such as emission maximum, quantum yield, and lifetime were significantly affected by solvent polarity changes (Table 1). In water, with excitement at 388 nm, PNA base analogue **5** exhibited an emission band at 528 nm with a quantum yield of 0.13 and lifetime of 1.16 ns (Figures 2 A and S1 B). As the solvent polarity was progressively reduced by using water/dioxane mixtures, a substantial blue shift in emission maximum (528 to 463 nm) was observed. In the least polar solvent (dioxane), the PNA base displayed a very high quantum yield (0.62) and long lifetime (2.46 ns) relative to those in water. A linear correlation between Stokes shift observed in different solvents and the Reichardt microscopic solvent polarity parameter *E*_T_ *(30)* confirmed the microenvironment‐sensitivity of **5** (Figure 2 B).^30^ Interestingly, the changes in intensity, quantum yield, and lifetime were found to be nonlinear as the polarity of the medium was changed from water to water/dioxane mixtures to dioxane. In the PNA base analogue **5**, the naphthalimide core can potentially rotate about the ethynyl linker, and hence its fluorescence should depend on the relative conformation of the naphthalimide and uracil rings, which can in turn be influenced by the viscosity of the medium. Because the water/dioxane mixtures also exhibit small difference in viscosity (Table 1), the observed fluorescence properties of **5** in different media are due to a combined effect of polarity and viscosity.^31^

**Table 1:** Photophysical properties of fluorescent PNA base analogue **5** in various solvents.

Solvent	*λ*_max_^[a]^	*λ*_em_	*Φ* ^[b]^	*τ*_av_^b]^	*E*_T_(30)	Viscosity
	[nm]	[nm]		[ns]	[kcal mol^−1^]	(cp)
water	388	528	0.13	1.16	62.8	0.89
25 % dioxane	395	522	0.34	2.51	57.4	1.95
50 % dioxane	398	520	0.47	3.07	53.2	1.65
75 % dioxane	399	509	0.65	3.46	49.6	1.33
dioxane	401	463	0.62	2.46	36.0	1.17

[a] Lowest‐energy maximum is given. [b] Standard deviations for quantum yield (*Φ*) and average lifetime (*τ*_av_) are ≤0.01 and ≤0.05 ns, respectively. Samples for absorption (25 μm) contained 2.5 % DMSO and samples for fluorescence (5 μm) contained 0.5 % DMSO.

### Responsiveness of emissive PNA analogue 5 in different neighboring base environments

**Figure 3:**
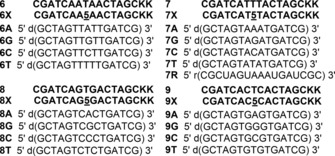
Sequences of unmodified PNA oligomers **6**–**9** and naphthalimide‐modified PNA oligomers **6 X**–**9 X** prepared by SPPS. PNA sequences (highlighted in bold) are each written from N to C terminus. Custom DNA and RNA ONs (“d” and “r”, respectively, preceding the parentheses) used in this study are also shown.

Application of fluorescent nucleobase analogue probes in nucleic acid studies is largely empirical and depends on the fluorescence of the probe in different nucleobase environments. A series of 15‐mer PNA oligomers **6 X**–**9 X**, containing fluorescent PNA base **5** in‐between different purine and pyrimidine bases, was synthesized by SPPS protocols to study the responsiveness of the probe in different nucleobase environments (Figure 3). The aqueous solubility of PNA oligomers was enhanced by adding two lysine residues at the C termini. The integrity of synthesized PNAs was ascertained by MALDI‐TOF mass analysis (Figure S2 and Table S1).

The PNA oligomers were then hybridized with a set of DNA ONs such that the emissive analogue **5** was placed opposite to perfect complementary or mismatched bases (Figure 3). Circular dichroism (CD) analysis of control unmodified and fluorescently modified PNA**⋅**DNA duplexes indicated that the modification had only a minor impact on the duplex structure (Figure S3). Although thermal melting experiments indicated that the modification had a noticeable destabilization effect on the duplex, the Lucifer‐modified PNA oligomers formed stable hybrids with complementary ONs at RT and under the conditions used for fluorescence analysis (Table S2).

**Figure 4:**
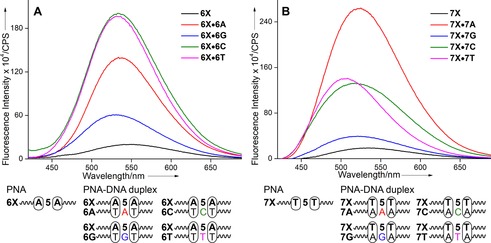
A) Emission spectra (0.5 μm) of emissive PNA oligomer **6 X** and PNA**⋅**DNA duplexes incorporating **6 X**. B) Emission spectra (0.5 μm) of emissive PNA oligomer **7 X** and PNA**⋅**DNA duplexes incorporating **7 X**. Samples were prepared in 10 mm phosphate buffer (pH 7.1, 100 mm NaCl, 0.1 mm EDTA) and excited at 400 nm. Excitation and emission slit widths were kept at 6 and 8 nm, respectively.

The emissive analogue placed in‐between adenine residues in PNA **6 X** showed a weak emission band around 548 nm (Figure 4 A). Upon hybridization with cDNA ONs, significant enhancement in fluorescence intensity accompanied by small changes in emission maximum was observed. Emissive base **5**, when located opposite to dT and dC residues and sandwiched between A**⋅**dT pairs in PNA**⋅**DNA duplexes **6 X⋅6 T** and **6 X⋅6 C**, displayed significant enhancement in intensity in comparison with when it was placed opposite to purine bases dA and dG (**6 X⋅6 A** and **6 X⋅6 G**). Interestingly, PNA**⋅**DNA duplex **7 X⋅7 A**, in which the fluorescent PNA base **5** is base‐paired with dA and flanked by T**⋅**dA pairs, displayed significant enhancement in fluorescence relative to when base **5** is placed opposite to dT, dC, and dG mismatches (**7 X⋅7 T**, **7 X⋅7 C** and **7 X⋅7 G**, Figure 4 B). However, in the cases of heteroduplexes made of PNA oligomers **8 X** and **9 X**, in which the modification is sandwiched between G**⋅**dC or C**⋅**dG pairs, the overall fluorescence was very low (Figure S4). Barring a few exceptions,^32^ fluorescence of probes located in the vicinity of guanine residues is well known to be quenched by photoinduced electron transfer processes, so this observation is reasonable.^33^ Collectively, these results indicate that the PNA base analogue is responsive to its neighboring nucleobase environment. Further, PNA**⋅**RNA duplex **7 X⋅7 R**, made by hybridizing PNA**⋅7 X** with complementary RNA ON **7 R**, also showed significant enhancement in fluorescence intensity (≈12‐fold) relative to the single‐stranded PNA **7 X** (Figure S4 C). As might be expected, the PNA**⋅**RNA duplex **7 X⋅7 R** was found to be more stable than the PNA**⋅**DNA duplex **7 X⋅7 A** (Figure S3 and Table S2). The enhancement in fluorescence intensity displayed by PNA**⋅**DNA/RNA ON duplexes could be due to the following reasons. A considerable blue shift in the emission band (≈520 nm) in the cases of the PNA**⋅**DNA and PNA**⋅**RNA duplexes **7 X⋅7 A** and **7 X⋅7 R**, respectively, indicates that the fluorophore is in a more nonpolar environment than in the single‐stranded PNA **7 X** (530 nm). It was known from the photophysical studies (Table 1) that the Lucifer‐modified PNA base analogue is more emissive in a nonpolar environment. Further, in the base‐paired state, the Lucifer chromophore attached through a rotatable ethynyl linker to the uracil ring could be rigidified, leading to an enhancement in fluorescence.^31^ A negative impact of base pairing would be that the modified base might experience partial stacking interaction with adjacent bases, which could lower the extinction coefficient and hence the fluorescence, according to Strickler–Berg theory.^34^ Hence, it is likely that the nonpolar environment and the rigidification of the fluorophore would have resulted in the overall enhancement of fluorescence in the duplex state. Taken together, the high fluorescence of PNA base **5**, when located in the vicinity of a triplet adenine in an RNA ON sequence, and the higher stability exhibited by PNA**⋅**RNA duplex prompted us to design a PNA oligomer probe to image poly(A)‐containing nucleic acids in cells.

### Imaging poly(A) RNAs in cells with the aid of a 5‐labeled PNA probe

**Figure 5:**
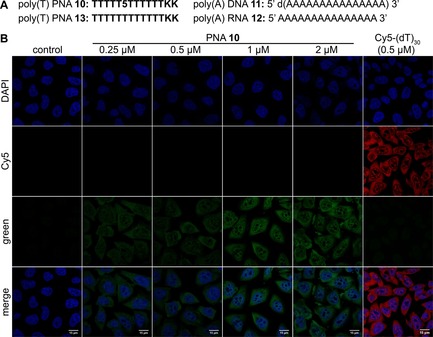
A) Sequences of poly(T) PNA probe **10**, containing fluorescent PNA base **5**, and of model poly(dA) and poly(A) ONs **11** and **12**, respectively [**13** is control unmodified poly(T) PNA oligomer]. B) Imaging cellular poly(A) RNAs with PNA **10** (green) and DNA ON Cy5‐(dT)_30_ (red). Cultured DLD1 cells were fixed, permeabilized, and hybridized with increasing concentrations of PNA **10** (0.25–2.0 μm) or Cy5‐(dT)_30_ (0.5 μm).

In order to set up a fluorescence microscopy assay to image cellular poly(A) RNAs, the 12‐mer poly(T) PNA probe **10** containing the fluorescent base **5** was prepared (Figure 5 A). The poly(T) PNA **10** exhibited a significantly higher quantum yield (0.22±0.01) than the free PNA base analogue **5** in water (0.13±0.01, Table 1). It is interesting to note that PNA **10** is intrinsically more fluorescent than PNA **7 X**; this is likely due to the differences in the microenvironment. PNA **7 X** exhibits a red‐shifted emission maximum (≈530 nm) in comparison with PNA **10** (515 nm). This indicates that the Lucifer chromophore in PNA **7 X** is in a more polar environment than in PNA **10** (Table 1). Hence, the stronger fluorescence of PNA **10** is consistent with a nonpolar environment being experienced by the fluorophore. Further, the influence on fluorescence properties is not just limited to the immediate nucleobase environment but also depends on the sequence, which supports the secondary structure, and hence the environment around the fluorophore.

The PNA probe was hybridized to model poly(dA) DNA ON and poly(A) RNA ON **11** and **12**, respectively. A duplex of poly(T) PNA **10** and poly(A) RNA ON **12** (**10⋅12**) displayed a significantly higher quantum yield (0.29±0.01) than the equivalent PNA**⋅**DNA duplex **10⋅11** (0.17±0.01).

The utility of emissive PNA **10** for detecting a poly(A) tail in a longer RNA transcript (≈1 kb) was then explored. RNA transcript **14**, with a 3′‐poly(A) tail (35 adenosine residues), and control transcript **15**, without a poly(A) tail, were synthesized by in vitro transcription with use of the appropriate plasmids (Figure S5 A). Incubation of PNA **10** with RNA **14**—containing a poly(A) tail—at 37 °C in a hybridization buffer typically used for imaging RNA in cells resulted in a discernible enhancement in fluorescence intensity relative to ssPNA **10** (Figure S5 B). Under similar conditions, incubation of **10** with RNA **15** produced only a minor change in fluorescence intensity. Encouraged by these results, we studied the efficacy of PNA **10** as a hybridization probe for imaging cellular poly(A) RNAs in cultured human cancer cells (e.g., DLD1 and HeLa).

DLD1 (human colon cancer) cells cultured on coverslips were fixed and permeabilized and incubated with increasing concentrations of poly(T) PNA **10** (0.25–2.0 μm) in a hybridization buffer at 37 °C for 2.5 h. Cells were counterstained with 2‐(4‐amidinophenyl)‐1H‐indole‐6‐carboxamidine (DAPI) and imaged with a confocal microscope. An RNase inhibitor (vanadyl ribonucleoside complex) was used in all steps to minimize the degradation of RNA. As little as 0.25 μm of the PNA probe produced visible nuclear and cytoplasmic staining (green), which was found to increase with increasing probe concentration (Figures 5 and S6). A characteristic punctate nuclear and uniform cytoplasmic staining, resembling the poly(A) RNA staining pattern reported in the literature, was observed (Figure S7).14b–14d As a positive control, cells were incubated with 5′‐Cy5‐(dT)_30_ (0.50 μm), a commercially available fluorescent DNA ON for detection of poly(A) RNAs.14b, 14c It showed a staining pattern (red) similar to that produced by the naphthalimide‐modified PNA probe **10** (Figure 5). Cells not treated with PNA/DNA probe did not show any fluorescence signal from red or green channels.

**Figure 6:**
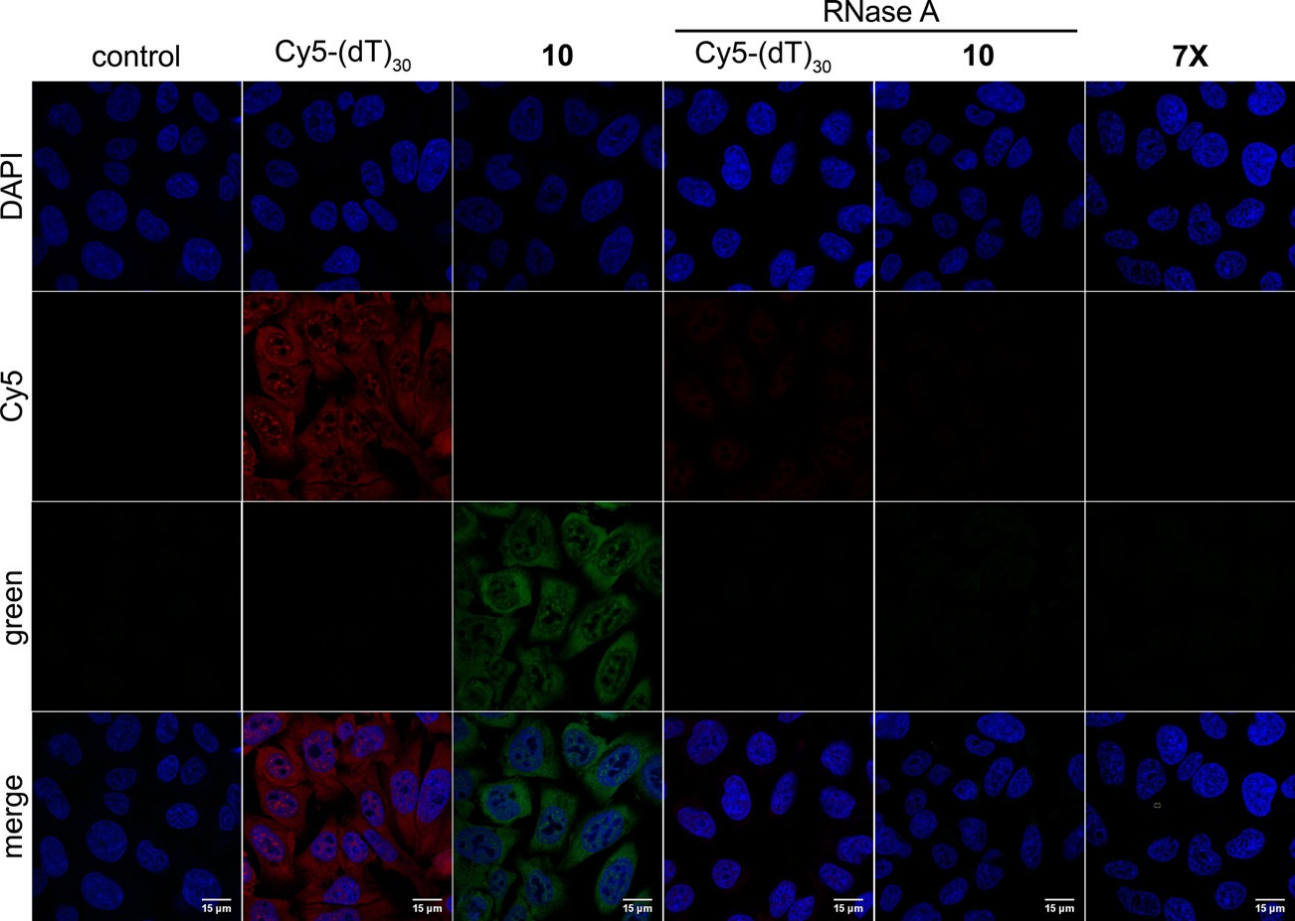
Poly(T) PNA probe **10** and Cy5‐(dT)_30_ label poly(A) RNAs. Cultured DLD1 cells were treated with RNase A and then subjected to hybridization with PNA **10** (1.0 μm) and Cy5‐(dT)_30_ (0.5 μm) probes (columns 4 and 5). A random sequence of naphthalimide‐modified PNA oligomer **7 X** (1.0 μm) did not stain poly(A) RNAs (column 6).

Cells were then incubated with RNase A and subjected to hybridization with fluorescent PNA **10** or Cy5‐(dT)_30_. The RNase treatment almost completely eliminated the fluorescence signal from the cells incubated with PNA or ON probe (Figure 6 and Figure S6). RNase A degrades the RNA from the 3′‐end, thereby removing poly(A) stretches, and hence depletes the signal.

Further, staining experiments were performed in the presence of a known polymerase inhibitor (actinomycin D), which would reduce the formation of polyadenylated mRNAs.^35^ Cells in culture treated with the inhibitor and then hybridized with Cy5‐(dT)_30_ or poly(T) PNA probe **10** exhibited noticeably reduced fluorescence (Figure S8). Cells treated with a random sequence of naphthalimide‐modified PNA oligomer **7 X** did not stain cellular RNA (Figure 6). These results suggest that the probes bind to poly(A) RNAs in cells and provide a means to visualize them by fluorescence microscopy.

### Lucifer‐labeled poly(T) PNA probe 10 binds to poly(A) RNAs more effectively than Cy5‐(dT)_30_ DNA ON

**Figure 7:**
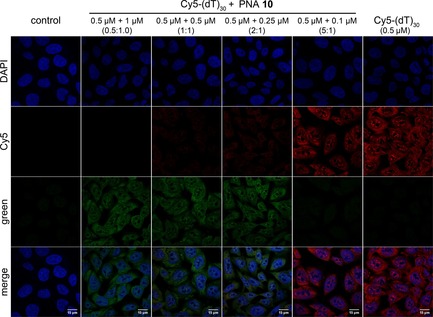
Competition assay indicates that PNA probe **10** binds to poly(A) RNAs with higher affinity than Cy5‐(dT)_30_. Cultured DLD1 cells were fixed, permeabilized, and hybridized with increasing molar ratios of Cy5‐(dT)_30_ to PNA **10**.

A competition assay to study the relative binding affinity of Cy5‐(dT)_30_ and poly(T) PNA probe **10** to cellular poly(A) RNAs was carried out by performing the hybridization step with increasing molar ratios of DNA to PNA. Even at 2 equivalents of the DNA ON probe, the cells exhibited fluorescence predominantly from the green channel, due to the effective binding of the PNA probe to poly(A) RNAs (Figure 7). As much as 5 equivalents of Cy5‐(dT)_30_ were required to overcome the binding affinity of the PNA to poly(A) RNAs and to produce a signal in the red channel. In a similar experiment, cells incubated with a mixture of 12‐mer unmodified poly(T) PNA **13** and Cy5‐(dT)_30_ did not exhibit fluorescence as a result of effective binding of the non‐fluorescent PNA to poly(A) RNAs (Figure S9). However, in the presence of a fluorescent PNA **7 X** of a random sequence, the staining ability of Cy5‐(dT)_30_ was not significantly affected (Figure S9).

The fluorescent poly(T) PNA **10** was also found to be highly efficient in staining poly(A) RNA sequences in HeLa cells (Figures S10 and S11). These results demonstrate that the Lucifer‐labeled PNA probe binds strongly and with high specificity to poly(A) RNAs, thereby offering a robust assay for imaging of endogenous RNAs in cells. This notion was further supported by *K*_d_ and *T*_m_ measurements. The *K*_d_ values of Lucifer‐labeled poly(T) PNA**⋅**poly(A) RNA duplex **10⋅12** and of Cy5‐labeled poly(T) DNA**⋅**poly(A) RNA duplex Cy5‐(dT)_30_ **⋅12** were determined by fluorescence and gel‐shift assays, respectively (Figure S12). The results revealed that the Lucifer‐labeled poly(T) PNA **10** [*K*_d_=(0.22±0.01) μm] has a significantly higher binding affinity for poly(A) RNA than Cy5‐(dT)_30_ [*K*_d_=(0.99±0.17) μm]. The PNA**⋅**RNA duplex **10⋅12** also exhibited a significantly higher *T*_m_ value [*T*_m_=(75.3±0.7) °C] than the DNA**⋅**RNA duplex Cy5‐(dT)_30_⋅**12** [*T*_m_=(37.2±0.6) °C].

## Conclusion

We have introduced the new base‐modified fluorescent PNA building block **5**, incorporating the Lucifer chromophore, which is highly responsive to its neighboring base environments in PNA**⋅**ON duplexes. The compatibility of emissive PNA base **5** with conventional confocal microscope and the ability of **5**‐labeled poly(T) PNA probe **10** to report a specific RNA sequence through fluorescence enabled the development of a robust method for imaging of endogenous poly(A) RNAs in cells. Importantly, the short poly(T) PNA probe of 12 bases targeted against poly(A) RNAs displayed significantly stronger binding than the commercially available DNA ON hybridization probe Cy5‐dT_30_. Taken together, our results suggest that microenvironment‐sensitive nucleobase analogues incorporated into PNAs, rather than ONs, could serve as useful hybridization probes for imaging nucleic acid sequences in cells. We are in the process of developing multichromophoric PNA oligomers incorporating adjacent Lucifer dye units or combinations of fluorophores to enhance the spectral properties of the PNA probes. Such multichromophoric systems at the ON level have shown interesting photophysical phenomena, yielding fluorescence properties such as large Stokes shifts, high quantum yields, and emission wavelengths throughout the visible region.^36^

## Experimental Section

Detailed procedures for the synthesis of the fluorescent PNA base analogue and its fluorescence analysis and incorporation into PNA oligomers by solid‐phase methods are provided in the Supporting Information.

**Thermal melting and CD analysis of PNA duplexes**: The PNA**⋅**DNA and PNA**⋅**RNA duplexes were assembled by heating 1:1 mixtures of PNA and DNA/RNA oligomers (10.0 μm) in phosphate buffer [pH 7.1, 10 mm, NaCl (100 mm), EDTA (0.1 mm)] at 90 °C for 3 min. All PNA oligomers were pre‐warmed to 60 °C for 2 min to avoid self‐aggregation before preparation of samples. Duplex samples were allowed to cool slowly to RT and kept in an ice bath for ≈1 h. Samples were further diluted with phosphate buffer to give final duplex concentrations of 1.0 μm for *T*_m_ and 5.0 μm for CD analysis, respectively. UV‐thermal melting analysis of PNA duplexes was performed at least in duplicate by using a quartz cuvette and a Cary 300Bio UV/Vis spectrophotometer. The temperature was increased from 20 to 90 °C at 1 °C min^−1^, and the absorbance at 260 nm was measured every 1 °C interval. CD spectra of PNA duplexes were recorded with an average of three scans from 350 to 200 nm by using a quartz cuvette (Starna Scientific, path length 2 mm) and a JASCO J‐815 CD spectrometer at 20 °C and a scan speed of 100 nm min^−1^. All CD spectra were corrected by use of an appropriate blank solution in the absence of duplex.

**Fluorescence of model 15‐mer naphthalimide‐modified PNA oligomers 6 X⋅9 X and their PNA⋅DNA/RNA ON duplexes**: PNA**⋅**DNA/RNA ON duplexes were obtained by heating 1:1 mixtures of PNA and DNA/RNA (5.0 μm) in phosphate buffer [pH 7.1, 10 mm, NaCl (100 mm), EDTA (0.1 mm)] at 90 °C for 3 min. All PNA oligomers were pre‐warmed to 60 °C for 2 min to avoid self‐aggregation before preparation of samples. Samples were allowed to cool slowly to RT and kept in an ice bath for ≈1 h. Samples were further diluted with phosphate buffer to give final duplex concentrations of 0.5 μm. All samples were excited at 400 nm. Excitation and emission slit widths are provided in the figure captions. Fluorescence experiments were performed in duplicate in micro fluorescence cuvettes (Hellma, path length 1.0 cm) with Fluoromax‐4 spectrophotometers (Horibha Scientific).

**Quantum yield determination for poly(T) PNA 10 and its duplexes with DNA 11 and RNA 12**: The PNA**⋅**DNA/RNA duplexes **10⋅11** and **10⋅12** (0.5 μm) were prepared in phosphate buffer [pH 7.1, 10 mm, NaCl (100 mm), EDTA (0.1 mm)] by the procedure described above, and samples were subjected to steady‐state fluorescence analysis. Samples were excited at 400 nm, and excitation and emission slit widths were kept at 2 and 3 nm, respectively. The quantum yields of fluorescently modified PNA **10** and its duplexes **10⋅11** and **10⋅12** were determined relative to the quantum yield of PNA analogue **5** in phosphate buffer [pH 7.1, 10 mm, NaCl (100 mm), EDTA (0.1 mm)]. See the Supporting Information for more details.

**Synthesis of longer RNA transcripts 14 [with a 3′‐poly(A) tail] and 15 [without a poly(A) tail] by in vitro transcription**: RNA transcript **14**, with a 3′‐poly(A) tail (35 adenosine residues), and control RNA transcript **15**, without 3′‐poly(A) tail, were synthesized by in vitro transcription with use of the appropriate plasmids (Figure S5). Appropriate primers were used to carry out PCR amplification of the templates, which were further used in in vitro transcription reactions to produce **14** and **15**. Templates (200 ng) were subjected to transcription in buffer [1×, Tris**⋅**HCl (50 mm), MgCl_2_ (8 mm), spermidine (2 mm), NaCl (25 mm), pH 8.0] containing GTP, CTP, ATP, and UTP (each 2 mm), dithiothreitol (DTT, 10 mm), RNase inhibitor (Ribolock, 1 U μL^−1^), and T7 polymerase (1 μL). The reaction mixtures were incubated at 37 °C for 4 h and subsequently treated with DNase1 (5 U μL^−1^) in DNase buffer [Tris**⋅**HCl (40 mm), MgCl_2_ (8 mm), DTT (5 mm), pH 7.5] at 37 °C for 30 min to remove the DNA templates. RNA transcripts **14** (1005 bases) and **15** (970 bases) were purified by use of RNAiso plus.

**Fluorescence detection of longer RNA transcript containing a poly(A) tail by using emissive poly(T) PNA 10**: Transcripts **14** and **15** were individually incubated with PNA **10** at 37 °C for 2.5 h in hybridization buffer {dextran sulfate (10 %, *w*/*v*), formamide (40 %, *v*/*v*), salmon sperm DNA (30 ng μL^−1^), vanadyl ribonucleoside complex (VRC, RNase inhibitor, 500 μm) prepared in SSC buffer [2×, sodium citrate (0.03 m), NaCl (0.3 m), VRC (500 μm), pH 7.4]. The final concentrations of RNA transcripts **14**, **15**, and PNA probe **10** were 75, 75, and 225 nm, respectively. Samples were excited at 400 nm, and excitation and emission slit widths were kept at 4 and 5 nm, respectively. Fluorescence experiments were performed in duplicate in micro fluorescence cuvettes (Hellma, path length 1.0 cm).

**Imaging cellular poly(A) RNAs**

**Cell culture**: DLD‐1 (human colon cancer cells ATCC CCL‐221) cells were cultured in RPMI1640 medium (Gibco by Life Technologies, 61870‐036) supplemented with fetal bovine serum (10 %, Gibco by Life Technologies, 10437028) and penicillin/streptomycin (Gibco by life technologies, 15070‐063) under humidified atmosphere containing CO_2_ (5 %) at 37 °C. Cells (0.1–0.3 million) were seeded on coverslips placed in a 12‐well plate. The cells were allowed to grow for nearly 48 h before hybridization. HeLa cells were cultured as above in Dulbecco's modified Eagle's medium (DMEM, Gibco by Life Technologies, 11965‐092).

**Fluorescence hybridization assay**: Cells grown on coverslips were washed with PBS [1×, 500 μL, Na_2_HPO_4_ (10 mm), KH_2_PO_4_ (1.8 mm), KCl (2.7 mm), NaCl (137 mm), VRC (500 μm), pH 7.4] and fixed in paraformaldehyde (500 μL, 4 %) containing VRC (500 μm) for 15 min. Subsequently, cells were permeabilized with chilled methanol (95 %) for 5 min. Cells were then washed with SSC buffer (2×, 500 μL) and prehybridized in hybridization buffer [50 μL, dextran sulfate (10 %, *w*/*v*), formamide (40 %, *v*/*v*), salmon sperm DNA (30 ng μL^−1^), VRC (500 μm) prepared in SSC (2×)] for 30 min at 37 °C in an incubator. After prehybridization, cells were washed with SSC buffer (2×, 500 μL) and incubated either in Cy5‐(dT)_30_ (0.5 μm) or in increasing concentrations of PNA probe **10** (0.25–2.0 μm) in hybridization buffer for 2.5 h at 37 °C. Cells were then washed with SSC (2×, 500 μL and 0.1×, 500 μL). Cells were counterstained with DAPI [55 nm, 500 μL in SSC (2×)] for 3 min and washed with SSC (0.1×, 500 μL). Coverslips were then placed on a microscope slide with anti‐fade mounting medium (7 μL) and sealed with nail polish. Finally, cells were imaged with a Confocal Laser Scanning Microscope (oil immersion, 40× lens).

**RNase A treatment**: Cells were incubated in RNase A [500 μL, 0.5 μg mL^−1^ in PBS (1×)] solution for 1 h at 37 °C. Cells were then washed with SSC (2×, 500 μL) and subjected to prehybridization in hybridization buffer. Finally, cells were incubated either with Cy5‐(dT)_30_ (0.5 μm) or PNA probe **10** (1.0 μm) in hybridization buffer for 2.5 h at 37 °C. Cells were imaged as described above.

**Competition assay**: Cells were incubated with increasing molar ratios of Cy5‐(dT)_30_ (0.5 μm) to PNA **10** (1.0, 0.5, 0.25, 0.1 μm) in hybridization buffer for 2.5 h at 37 °C. In control experiments, cells were incubated with a mixture of Cy5‐(dT)_30_ (0.5 μm) and either random naphthalimide‐modified PNA **7 X** (1.0 μm) or control unmodified poly(T) PNA **13** in hybridization buffer for 2.5 h at 37 °C. Cells were washed, counterstained with DAPI, and imaged as above.

**Quantification of poly(A) RNA‐stained cells**: Integrated densities of poly(A) RNA‐stained cells were obtained by use of ImageJ software. Images were acquired for a given cover slip, and ≈100 cells were counted from three independent biological replicates. Normalized integrated density for PNA probe **10** (green) and Cy5‐(dT)_30_ (red) upon binding to poly(A) RNA in a particular cell was obtained by subtracting the integrated density of background from the integrated density of the given cell. Scatter plots were generated with GraphPad Prism software, and statistical significance was calculated by using the Mann–Whitney U test (Figure S6).

## Conflict of interest

*The authors declare no conflict of interest*.

## Supporting information

As a service to our authors and readers, this journal provides supporting information supplied by the authors. Such materials are peer reviewed and may be re‐organized for online delivery, but are not copy‐edited or typeset. Technical support issues arising from supporting information (other than missing files) should be addressed to the authors.

Supplementary
