# Supplementary material for: A Lucifer‐Based Environment‐Sensitive Fluorescent PNA Probe for Imaging Poly(A) RNAs
Source: Chembiochem. 2018 Mar 13;19(8):826–35. doi: 10.1002/cbic.201700661 (PMC5972818; doi:10.1002/cbic.201700661)
Supplement: Supplementary file 1 — Supplementary [file CBIC-19-826-s001.pdf]

CHEMBIOCHEM

Supporting Information

A Lucifer-Based Environment-Sensitive Fluorescent PNA Probe for Imaging Poly(A) RNAs

Pramod M. Sabale, Uddhav B. Ambi, and Seergazhi G. Srivatsan*[a]

cbic_201700661_sm_miscellaneous_information.pdf

Supporting Information

Contents	Page #
**1. Materials**	S2
**2. Instrumentation**	S2
**3. Synthesis of fluorescent PNA monomer**	S3
**Scheme S1.** Synthesis of fluorescent naphthalimide-modified uracil *aeg* PNA monomer **4** and **5**.	S3
**Scheme S2.** Synthesis of Fmoc-protected naphthalimide-conjugated uracil PNA monomer.	S5
**4. Photophysical characterization of PNA base analog 5**	S7
**5. Quantum yield determination of emissive PNA base analog 5**	S7
**Figure S1.** (**A**) Absorption spectra of PNA analog **5** (25.0 μM) in various solvents. (**B**) Excited-state decay profile (5.0 μM) of free PNA analog **5** in solvents of different polarity.	S7
**6. Solid-phase synthesis of control unmodified and naphthalimide-modified PNA oligomers**	S8
**7. HPLC analysis of PNA oligomers**	S9
**8. MALDI-TOF mass measurement of PNA oligomers**	S9
**Figure S2.** (**A**) Representative RP-HPLC chromatogram of fluorescently-modified PNA oligomer. (**B**) Representative MALDI-TOF mass spectrum of fluorescently-modified PNA oligomer.	S9
**Table S1.** ε260 and MALDI-TOF mass analysis of PNA oligomers.	S9
**Figure S3.** CD spectra and UV-thermal melting profile of control-unmodified and fluorescently-modified PNA-ON duplexes.	S10
**Table S2.** T_m values of control unmodified and fluorescently-modified PNA-ON duplexes.	S10
**Figure S4.** (**A**) Emission spectra of PNA oligomers and their duplexes	S11
**Figure S5.** (**A**) Sequence of the longer RNA transcript **14** containing a 3′ poly(A) tail and control RNA transcript **15** without 3′ poly(A) tail. (**B**) Fluorescence spectrum of PNA **10** and PNA **10** incubated with RNA transcripts **14** and **15**.	S12
**Figure S6.** Scatter plots showing normalized integrated density for poly(A) signals upon binding of PNA probe **10** and Cy5-$(dT)_{30}$.	S13
**Figure S7.** Magnified confocal images of poly(A) RNAs-stained DLD1 cells using Cy5-$(dT)_{30}$ (0.5 μM) and poly(T) PNA **10** (1.0 μM).	S13
**Figure S8.** Effect of polymerase inhibitor on the poly(A) RNA staining by poly(T) PNA **10** and Cy5-$(dT)_{30}$.	S14
**Figure S9.** Competition assay between Cy5-$(dT)_{30}$ and unmodified poly(T) PNA **13**, and Cy5-$(dT)_{30}$ and fluorescent PNA oligomer (**7X**) of a random sequence.	S15
**Figure S10.** Imaging cellular poly(A) RNAs using DNA ON Cy5-$(dT)_{30}$ and PNA probe **10** in HeLa cells.	S16
**Figure S11.** Competition assay indicates that PNA probe **10** binds to poly(A) RNAs of HeLa cells with higher affinity as compared to Cy5-$(dT)_{30}$.	S17
**9. Binding assay: binding of poly(T) PNA 10 and Cy5-$(dT)_{30}$ to poly(A) RNA ON 12**	S17
**Figure S12.** Curve fits for the binding of poly(T) PNA **10** or Cy5-$(dT)_{30}$ to poly(A) RNA	S18
**10. NMR Spectra**	S19
**11. Reference**	S26

1. Materials

Chloroacetyl chloride, di-*t*-butyl dicarbonate, ethane-1,2-diamine, ethyl bromoacetate, TFA, piperidine, EDC and silica gel (100-200 mesh) were purchased from Spectrochem, India. Dry DMF and dry dichloromethane were purchased from Rankem, India. Acetic anhydride was purchased from Thomas Baker, India. 5-Iodouracil, sodium ascorbate, propargylamine, tetrakis (triphenylphosphine) palladium (0), pyridine, *N*-hydroxybenzotriazole (HOBt), 3-[bis(dimethylamino)methyliumyl]-3*H*-benzotriazol-1-oxide hexafluorophosphate (HBTU), *N*,*N*-diisopropylethylamine (DIPEA), 1,2-ethanedithiol, thioanisole, anisole, Fmoc-Lys(Boc)-OH and trifluoromethanesulfonic acid (TFMSA), vanadyl ribonucleoside complex (VRC, RNase inhibitor) solution, paraformaldehyde, dextran sulphate, formamide, salmon sperm DNA, RNase A and DAPI were purchased from Sigma-Aldrich. Chemicals for preparing buffer solutions were obtained from Sigma-Aldrich. 5-Iodouracil PNA ester **1**[S1] and 4-ethynyl-1,8-naphthalimide derivative **2**[S2] were synthesized by using our reported procedure. Boc-and Fmoc-protected *aeg*-PNA monomers and Fmoc PNA backbone **d** were purchased from ASM Research Chemicals. 4-Methylbenzhydrylamine•HCl (MBHA) resin LL (100-200 mesh) and Boc-Lys(2-Cl-Z)-OH were obtained from Novabiochem. Rink amide AM resin (100-200 mesh) was purchased from GL Biochem (Shanghai) Ltd. DNA ONs were purchased from Integrated DNA Technologies. Custom synthesized RNA ON purchased from Dharmacon RNAi Technologies was deprotected according to the supplier's procedure. Reagents for cell culture experiments such as RPMI1640 and DMEM medium (gibco®), fetal bovine serum (gibco®) and penicillin streptomycin (gibco®) and anti-fade mounting media were obtained from Life Technology, India. 5′-Fluorescent Cy5 oligo-dT probe (Cy5-$(dT)_{30}$) was purchased from Sigma-Aldrich. ATP, GTP, CTP, UTP, RNase inhibitor (Ribolock) were purchased from Thermo Scientific. Taq DNA polymerase (Cat#RR310Q), DNase-1 enzyme (Cat#2270A) and RNAiso plus reagent (Cat#9108) were obtained from Takara™ Clontech. Isopropanol (Cat#13825), chloroform (Cat#22465) for RNA purification was bought from Fisher Scientific. Agarose SeaKem® LE Agarose (Cat#50004) was purchased from Lonza. Gel purification and PCR clean-up kit (Cat#740609.50) were purchased from Macherey Nagel. All ONs were purified by polyacrylamide gel electrophoresis (PAGE) under denaturing conditions and desalted using Sep-Pak Classic C18 cartridges (Waters Corporation). Autoclaved water was used in all biochemical reactions and fluorescence measurements.

2. Instrumentation

NMR spectra were recorded on a 400 MHz Jeol ECS-400 spectrometer (^{1}H 400 MHz and ^{13}C 100 MHz) and processed in Mnova NMR software from Mestrelab Research. Mass measurements were recorded on an Applied Biosystems 4800 Plus MALDI-TOF/TOF analyzer instrument and Water Synapt G2 High Definition mass spectrometers. Reverse-phase (RP) flash chromatography (C18 RediSepRf column) purifications were carried out using Teledyne ISCO, Combi Flash Rf. Microwave reactions were performed using CEM discover microwave synthesizer. PNA oligomers were purified by using Agilent Technologies 1260 Infinity HPLC. Absorption spectra were recorded on a Shimadzu UV-2600 spectrophotometer. UV-thermal melting analysis of duplexes was performed on a Cary 300Bio UV-Vis spectrophotometer. Steady-state fluorescence experiments were carried out in a microfluorescence cuvette (Hellma, path length 1.0 cm) on a Fluoromax-4 and Fluorolog-3 spectrophotometer (Horibha Scientific). Time-resolved fluorescence experiments were carried out on a TCSPC instrument (Horiba JobinYvon, Fluorolog 3). All CD spectra were recorded on JASCO J-815 CD spectrometer. Confocal imaging was carried out on a Zeiss LSM710 Confocal Laser Scanning Microscope with oil immersion using 40X lens.

Confocal images were acquired by using the following excitation and emission settings. DAPI (λ_{ex} = 405 nm and λ_{em} = 420–476 nm, blue channel); naphthalimide-modified poly(T) PNA probe **10** (λ_{ex} = 405 nm and λ_{em} = 510–580 nm, green channel); Cy5-(dT)$_{30}$ probe (λ_{ex} = 633 nm and λ_{em} = 640–760 nm, red channel). The Zen 2012 software was used for image acquisition. Java Image J software was used to process the images acquired from the instrument.

3. Synthesis of fluorescent PNA monomer

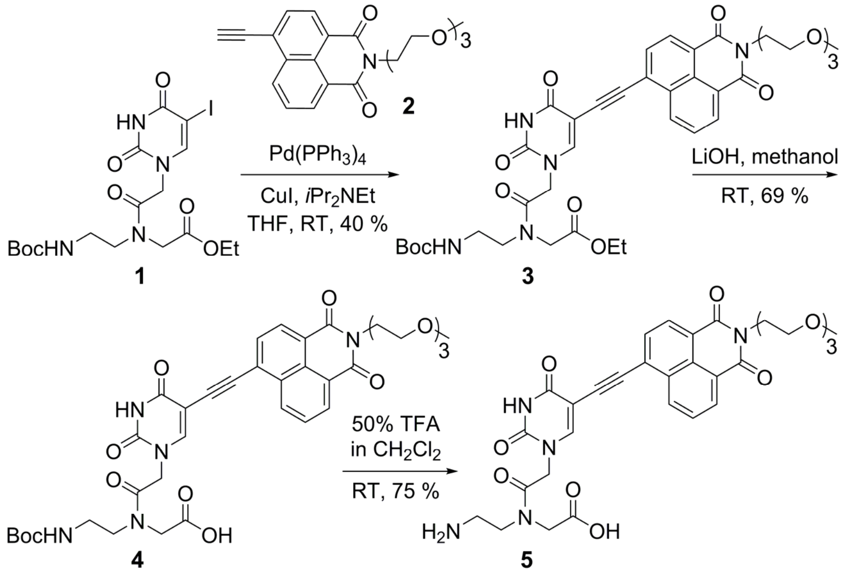

Scheme S1. Synthesis of fluorescent naphthalimide-modified uracil *aeg* PNA monomer **4** used in the solid-phase PNA synthesis (SPPS). Fully deprotected analog **5** was used in the photophysical analysis.

Ethyl *N*-(2-((*tert*-butoxycarbonyl)amino)ethyl)-*N*-(2-(5-((2-(2-(2-(2-methoxyethoxy)ethoxy)ethyl)-1,3-dioxo-2,3-dihydro-1*H*-benzo[*de*]isoquinolin-6-yl)ethynyl)-2,4-dioxo-3,4-dihydropyrimidin-1(2*H*)-yl)acetyl)glycinate 3:

A mixture of 5-iodouracil PNA ester **1**[S1] (1.10 g, 2.10 mmol, 1.0 equiv.), CuI (80.0 mg, 0.42 mmol, 2.0 equiv.), tetrakis (triphenylphosphine) palladium(0) (0.24 g, 0.21 mmol, 0.1 equiv.) and 4-ethynyl-1,8-naphthalimide derivative **2**[S2] (0.77 g, 2.10 mmol, 1.0 equiv.) were dissolved in degassed anhydrous 27.5 mL THF under nitrogen atmosphere. DIPEA (1.28 mL, 7.35 mmol, 3.5 equiv.) was then slowly added, and the reaction mixture was stirred in the dark at RT for 12 h. Solvent was evaporated using rotary evaporator. The residue was dissolved in ethyl acetate and further washed with water. The organic layer was separated and dried over sodium sulphate. The residue obtained after evaporation was purified using silica gel Combi-flash column chromatography (methanol-dichloromethane solvent systems) to afford yellow solid **3** (0.63 g, 40%). TLC R_f = 0.48 ($CHCl_3$:MeOH = 9:1); ^{1}H-NMR (400 MHz, $CDCl_3$): δ = 8.73 (d, J = 8.4 Hz, 1H), 8.54 ($_{app}$t, J = 6.8 & 5.6 Hz, 1H), 8.42 ($_{app}$t, J = 7.6 & 5.2 Hz, 1H), 7.80–7.76 (m, 2H), 7.70 (br, 1H), 5.64 (ma.) and 5.07 (mi.) ($_{app}$t, J = 5.2 Hz, 1H), 4.76 (ma.) and 4.60 (mi.) (s, 2H), 4.40 (t, J = 6 Hz, 2H), 4.24 (m, 4H), 3.81 (t, J = 6 Hz, 2H), 3.71–3.69 (m, 2H), 3.62–3.60 (m, 2H), 3.58–3.56 (m, 4H), 3.45–3.42 (m, 2H), 3.37–3.34 (m, 2H), 3.30 (s, 3H), 1.46 (ma.) and 1.42 (mi.) (s, 9H), 1.34 (mi.) and 1.27 (ma.) (t, J = 7.2 Hz, 3H) ppm; ^{13}C-NMR (100 MHz, $CDCl_3$): δ = 169.7 (ma.) and 169.4 (mi.), 167.3

(mi.) and 166.9 (ma.), 164.1 (mi.) and 163.8 (ma.), 161.6, 156.2, 149.9, 148.5 (mi.) and 148.4 (ma.), 133.0, 132.3, 132.2, 131.8, 131.5, 130.3 (ma.) and 130.2 (mi.), 128.7, 128.6, 128.0 (mi.) and 127.8 (ma.), 127.1, 122.7 (ma.) and 122.2 (mi.), 99.7, 91.4, 90.2, 80.3, 72.0, 70.7, 70.6, 70.3, 68.0, 62.6, 62.0, 59.1, 49.4, 49.0, 48.6, 46.2, 39.3 (ma.) and 38.9 (mi.), 28.6 (ma.) and 28.5 (mi.), 14.2 ppm; HRMS: Calcd. for $C_{38}H_{45}N_5O_{12}Na$: 786.2962 $[M+Na]^+$; found: 786.2956.

***N*-(2-((*tert*-Butoxycarbonyl)amino)ethyl)-*N*-(2-(5-((2-(2-(2-(2-ethoxyethoxy)ethoxy)ethyl)-1,3-dioxo-2,3-dihydro-1*H*-benzo[*de*]isoquinolin-6-yl)ethynyl)-2,4-dioxo-3,4-dihydropyrimidin-1(2*H*)-yl)acetyl)glycine 4:**

To a solution of fluorescent PNA ester **3** (0.45 g, 0.59 mmol, 1.0 equiv.) in 13.5 mL methanol was added 6.75 mL 3% lithium hydroxide solution and the reaction mixture was stirred for 3 h at RT. The solvent was evaporated to dryness, and the residue was dissolved in water and washed with diethyl ether. To the aqueous extract was added saturated $KHSO_4$ solution to adjust the pH to ~4. The product was then extracted with EtOAc and solvent was evaporated. The residue was purified by reversed phase column chromatography (C18 Redi*SepRf* column, MeOH: H_2O) to afford the yellow solid **4** (0.3 g, 69%). TLC R_f = 0.12 (CH_2Cl_2:MeOH = 9:1); ^{1}H-NMR (400 MHz, d_6-DMSO): δ = 11.93 (br, 1H), 8.75 (d, J = 8.4 Hz, 1H), 8.54–8.26 (m, 3H), 8.00–7.88 (m, 2H), 7.64–7.55 (m, 2H), 7.00 (ma.) and 6.85 (mi.) (br, 1H), 4.82 (ma.) and 4.65 (mi.) (s, 2H), 4.24–3.99 (m, 4H), 3.67–3.41 (m, 13H), 3.14 (s, 3H), 1.39 (ma.) and 1.36 (mi.) (s, 9H) ppm; ^{13}C-NMR (100 MHz, d_6-DMSO): δ = 167.2 (mi.) and 166.6 (ma.), 163.2, 162.9, 161.9, 155.8 (ma.) and 155.6 (mi.), 151.2 (mi.) and 150.9 (ma.), 149.9, 132.1 (ma.) and 131.9 (mi.), 131.5, 131.3, 130.7 (ma.) and 130.6 (mi.), 130.2 (ma.) and 130.0 (mi.), 128.8 (ma.) and 128.7 (mi.), 128.1 (ma.) and 128.0 (mi.), 127.4 (ma.) and 127.3 (mi.), 126.5 (mi.) and 126.4 (ma.), 122.6 (ma.) and 122.5 (mi.), 121.5 (ma.) and 121.4 (mi.), 96.6, 92.3, 89.5, 78.1, 77.7, 71.2, 69.7, 69.6, 66.9, 58.0, 48.4, 47.9, 46.9, 38.0, 28.2 ppm; HRMS: Calcd. for $C_{36}H_{41}N_5O_{12}Na$: 758.2649 $[M+Na]^+$; found: 758.2657.

***N*-(2-Aminoethyl)-*N*-(2-(5-((2-(2-(2-(2-methoxyethoxy)ethoxy)ethyl)-1,3-dioxo-2,3-dihydro-1*H*-benzo[*de*]isoquinolin-6-yl)ethynyl)-2,4-dioxo-3,4-dihydropyrimidin-1(2*H*)-yl)acetyl)glycine 5:**

Solution of compound **4** (0.30 g, 0.41 mmol, 1.0 equiv.) in 50% TFA in dichloromethane (15 mL) was stirred for 3 h at RT. Reaction mixture was evaporated to dryness using NaOH trap, and residue was co-evaporated with dichloromethane (3 x 20 mL). Residue was further purified by RP-HPLC using a semipreparative column [conditions: 0−50% B in 10 min and 50−100% B in 10 min (Mobile phase A: 5% acetonitrile in H_2O containing 0.1% TFA. Mobile phase B: 100% acetonitrile in H_2O containing 0.1% TFA) and flow rate: 1 mL/min] to afford yellow solid **5** (0.13 g, 51%). ^{1}H-NMR (400 MHz, d_6-DMSO): δ = 11.99 (mi.) and 11.95 (ma.) (s, 1H), 8.81–8.69 (m, 1H), 8.59–8.54 (m, 1H), 8.46–8.30 (m, 2H), 7.99–7.77 (m, 5H), 5.12–4.69 (m, 2H), 4.36–4.06 (m, 4H), 3.69–3.64 (m, 3H), 3.57–3.53 (m, 3H), 3.48–3.45 (m, 2H), 3.43–3.40 (m, 2H), 3.29–3.27 (m, 2H), 3.14 (s, 3H), 3.11–2.96 (m, 2H) ppm; ^{13}C-NMR (100 MHz, d_6-DMSO): δ = 170.9 (mi.) and 170.8 (ma.), 168.2, 167.0, 163.2 (mi.) and 162.9 (ma.), 161.9, 151.1, 150.0, 131.9, 131.3, 130.7, 130.2, 130.0, 128.2, 127.4, 126.4, 122.6, 121.6, 96.7 (mi.) and 96.6 (ma.), 92.4 (ma.) and 92.3 (mi.), 89.6 (mi.) and 89.5 (ma.), 71.2, 69.7, 69.6, 66.9, 58.0, 48.8 (mi.) and 48.5 (ma.), 47.8, 45.0, 36.9 ppm; HRMS: Calcd. for $C_{31}H_{34}N_5O_{10}$: 636.2306 $[M+H]^+$; found: 636.2318. λ_{max} (H_2O) = 388 nm; ε_{260} = 9680 $M^{-1}cm^{-1}$, ε_{388} = 17700 $M^{-1}cm^{-1}$.

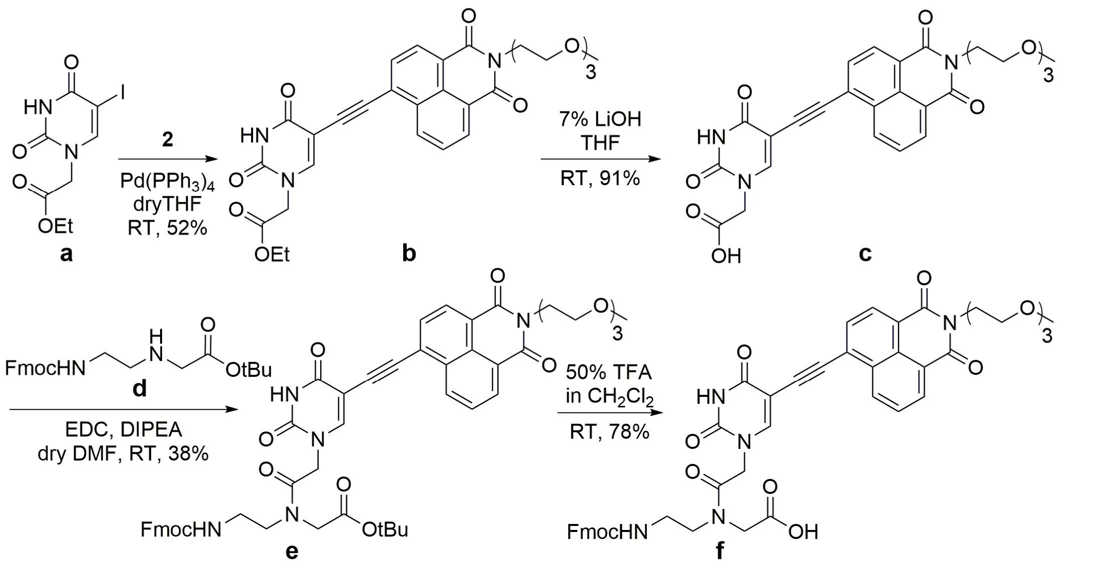

Scheme S2. Synthesis of Fmoc-protected naphthalimide-conjugated uracil PNA monomer **f** for SPPS by Fmoc-chemistry.

Ethyl 2-(5-((2-(2-(2-(2-methoxyethoxy)ethoxy)ethyl)-1,3-dioxo-2,3-dihydro-1*H*-benzo[*de*]isoquinolin-6-yl)ethynyl)-2,4-dioxo-3,4-dihydropyrimidin-1(2*H*)-yl)acetate b:
To a suspension of compound **a**[S3] (0.610 g, 1.88 mmol, 1.0 equiv.), **2** (0.680 g, 1.88 mmol, 1.0 equiv.), $Pd(Ph_3)_4$ (0.214 g, 0.19 mmol, 0.1 equiv.) and CuI (0.071 g, 0.38 mmol, 0.2 equiv.) in degassed dry THF (6 mL) was added *N*,*N*-diisopropylethylamine (1.13 mL, 6.58 mmol, 3.5 equiv.). The reaction mixture was stirred for 12 h at RT and filtered through Celite pad, washed with THF (2 x 10 mL). The filtrate was then evaporated and the residue was washed with petroleum ether (2 x 20 mL). The crude product was further re-crystallized using EtOAc (30 mL) to afford the product **b** as a yellow solid (0.545 g, 52%). TLC R_f= 0.41 (EtOAc); ^{1}H-NMR (400 MHz, d_6-DMSO): δ = 12.03 (br, 1H), 8.74 (d, *J* = 8.4 Hz, 1H), 8.53 (d, *J* = 7.2 Hz, 1H), 8.49 (s, 1H), 8.43 (d, *J* = 7.6 Hz, 1H), 7.98–7.92 (m, 2H), 4.65 (mi.) and 4.63 (ma.) (s, 2H), 4.25–4.17 (m, 4H), 3.66 (t, *J* = 6.4 Hz, 2H), 3.56–3.53 (m, 2H), 3.47–3.41 (m, 4H), 3.30–3.27 (m, 2H), 3.15 (s, 3H), 1.24 ($_{app}$t, *J* = 7.2 and 6.8 Hz, 3H) ppm; ^{13}C-NMR (100 MHz, d_6-DMSO): δ = 167.6, 163.1, 162.8, 161.7, 150.3, 149.8, 131.8, 131.2, 130.6, 130.1, 130.0, 128.0, 127.3, 126.2, 122.5, 121.6, 96.9, 92.0, 89.5, 71.1, 69.6, 69.5, 66.8, 61.4, 57.9, 49.0, 14.0 ppm; HRMS: (*m/z*): Calcd. for $C_{29}H_{29}N_3O_9Na$: 586.1801 $[M+Na]^+$; found: 586.1797.

2-(5-((2-(2-(2-(2-Methoxyethoxy)ethoxy)ethyl)-1,3-dioxo-2,3-dihydro-1*H* benzo[*de*]isoquinolin-6-yl)ethynyl)-2,4-dioxo-3,4-dihydropyrimidin-1(2*H*)-yl)acetic acid c:
To a solution of compound **b** (0.545 g, 0.97 mmol, 1.0 equiv.) in THF (11 mL), lithium hydroxide (7% solution in H_2O, 5.0 mL) was added dropwise and the reaction mixture was stirred for 2 h at RT. Reaction mixture was evaporated to dryness, and residue was dissolved in water (20 mL) and washed with diethyl ether (15 ml). To the aqueous extract was added saturated $KHSO_4$ solution (~5 mL) to adjust the pH to ~5 and product was precipitated out as yellow solid, which was further collected upon filtration as a yellow solid **c** (0.47 g, 91%). TLC R_f = 0.18 (CH_2Cl_2:MeOH = 8:2); ^{1}H-NMR (400 MHz, d_6-DMSO): δ = 11.98 (br, 1H), 8.72 (d, *J* = 8.4 Hz, 1H), 8.53–8.49 (m, 1H), 8.48 (s, 1H), 8.41 (d, *J* = 7.6 Hz, 1H), 7.97–7.87 (m, 2H), 4.53 (s, 2H), 4.22 (t, *J* = 6.4 Hz, 2H), 3.65 (t, *J* = 6.4 Hz, 2H), 3.55–3.53 (m, 2H),

3.47–3.41 (m, 4H), 3.30–3.27 (m, 2H), 3.14 (s, 3H) ppm; ^{13}C-NMR (100 MHz, d_6-DMSO): δ = 169.1, 165.7, 163.1, 162.8, 161.7, 150.6, 149.9, 131.8, 131.2, 130.6, 130.1, 129.9, 128.0, 127.3, 126.3, 122.5, 121.5, 96.7, 92.2, 89.4, 71.1, 69.6, 69.5, 66.9, 57.9, 49.1 ppm; HRMS: Calcd. for $C_{27}H_{25}N_3O_9Na$: 558.1488 [M+Na]$^+$; found: 558.1492.

***tert*-Butyl *N*-(2-((((9*H*-fluoren-9-yl)methoxy)carbonyl)amino)ethyl)-*N*-(2-(5-((2-(2-(2-(2-methoxyethoxy)ethoxy)ethyl)-1,3-dioxo-2,3-dihydro-1*H*-benzo[*de*]isoquinolin-6-yl)ethynyl)-2,4-dioxo-3,4-dihydropyrimidin-1(2*H*)-yl)acetyl)glycinate e:**
Solution of compound **c** (0.430 g, 0.80 mmol, 1.2 equiv.), Fmoc PNA backbone **d** (0.290 g, 0.67 mmol, 1.0 equiv.), EDC (0.153 g, 0.80 mmol, 1.2 equiv.), HOBt (0.108 g, 0.80 mmol, 1.2 equiv.) and DIPEA (0.23 mL, 1.34 mmol, 2.0 equiv.) in dry DMF (8.6 mL) were stirred for 4 h at RT under nitrogen atmosphere. Solvent was evaporated and residue was purified using silica gel chromatography with dichloromethane and methanol solvent system to afford yellow solid **e** (0.28 g, 38%). TLC R_f = 0.53 (EtOAc); ^{1}H-NMR (400 MHz, d_6-DMSO): δ = 11.94 (br, 1H), 8.73 (d, J = 8.4 Hz, 1 H), 8.55–8.48 (m, 1H), 8.40 (t, J = 7.0 Hz, 1H), 8.33 (ma.) and 8.30 (mi.) (s, 1H), 7.95–7.82 (m, 4H), 7.69–7.64 (m, 2H), 7.46–7.26 (m, 5H), 4.83 (ma.) and 4.65 (mi.) (s, 2H), 4.35–4.22 (m, 6H), 3.97 (s, 1H), 3.66 (t, J = 6.4 Hz, 2H), 3.56–3.53 (m, 2H), 3.47–3.40 (m, 6H), 3.29–3.27 (m, 4H), 3.14 (s, 3H), 1.48 (mi.) and 1.40 (ma.) (s, 9H) ppm; ^{13}C-NMR (100 MHz, d_6-DMSO): δ = 167.9, 166.7, 163.1, 162.8, 161.8, 157.4, 150.9, 149.9, 142.5, 139.4, 137.4, 131.9, 131.2, 130.6, 130.1, 129.9, 128.9, 128.0, 127.4, 127.2, 126.4, 122.5, 121.5, 121.3, 120.0, 109.7, 96.6, 92.4, 92.3, 89.4, 82.0, 81.0, 71.1, 69.6, 69.5, 66.9, 57.9, 54.9, 48.7, 48.4, 47.2, 38.8, 27.7 (ma.) and 27.6 (mi.) ppm; HRMS: (*m/z*): Calcd. for $C_{50}H_{51}N_5O_{12}Na$: 936.3432 [M+Na]$^+$; found: 936.3406.

***N*-(2-((((9*H*-Fluoren-9-yl)methoxy)carbonyl)amino)ethyl)-*N*-(2-(5-((2-(2-(2-(2-methoxyethoxy)ethoxy)ethyl)-1,3-dioxo-2,3-dihydro-1*H*-benzo[*de*]isoquinolin-6-yl)ethynyl)-2,4-dioxo-3,4-dihydropyrimidin-1(2*H*)-yl)acetyl)glycine f:**
Solution of compound **e** (0.13 g, 0.14 mmol, 1.0 equiv.) in 50% TFA in dichloromethane (15 mL) was stirred for 2 h at RT. Solvent was evaporated to dryness using NaOH trap, and residue was co-evaporated using dichloromethane (3 x 20 mL). Crude product was purified using silica gel column chromatography with dichloromethane and methanol solvent system to afford yellow product **f** (95 mg, 78%). TLC R_f= 0.33 (CH_2Cl_2:MeOH = 8.5:1.5); ^{1}H-NMR (400 MHz, d_6-DMSO): δ = 11.95 (mi.) and 11.90 (ma.) (br, 1H), 8.69–8.61 (m, 1 H), 8.48–8.24 (m, 3H), 7.84–7.47 (m, 7H), 7.39–7.26 (m, 4H), 4.83 (mi.) and 4.68 (ma.) (s, 2H), 4.33–4.13 (m, 5H), 3.99 (br, 2H), 3.66–3.63 (m, 2H), 3.55–3.53 (m, 2H), 3.47–3.45 (m, 3H), 3.43–3.41 (m, 4H), 3.31–3.27 (m, 3H), 3.15 (ma.) and 3.14 (mi.) (s, 3H) ppm; ^{13}C-NMR (100 MHz, d_6-DMSO): δ = 167.4, 166.5, 163.1, 162.8, 161.9, 156.4, 156.1, 151.1 (mi.) and 151.0 (ma.), 150.0, 143.8, 140.7, 140.6, 131.9, 131.2, 131.7, 130.6 (mi.) and 130.5 (ma.), 130.1, 130.0, 129.9, 129.8, 129.7, 128.0, 127.9, 127.8, 127.6 (mi.) and 127.5 (ma.), 127.3, 127.1, 126.4, 125.2, 120.1 (mi.) and 120.0 (ma.), 96.6, 92.5 and 92.4, 89.5, 71.2, 69.7, 69.6, 66.9, 65.6, 58.0, 51.2, 48.6, 46.8 (mi.) and 46.7 (ma.), 37.9 ppm; HRMS: Calcd. for $C_{46}H_{43}N_5O_{12}Na$: 880.2806 [M+Na]$^+$; found: 880.2799.

4. Photophysical characterization of PNA base analog 5

UV-Vis absorption: Samples of analog **5** (25.0 μM) were prepared in water, dioxane and their mixtures (25% dioxane, 50% dioxane, 75% dioxane in water). All solutions contained 2.5% DMSO.

Steady-state fluorescence: Emission spectra of analog **5** (5.0 μM) in water, dioxane and their mixtures (25% dioxane, 50% dioxane, 75% dioxane in water) were recorded by exciting the samples at respective longest absorption maximum (Table 1). Excitation and emission slit widths were kept at 1 and 3 nm, respectively. All solutions contained 0.5% DMSO.

Time-resolved fluorescence: Excited-state lifetime of Lucifer PNA analog **5** (5.0 μM) in various solvents was determined using TCSPC instrument (Horiba Jobin Yvon, Fluorolog-3). Samples were excited using a 371 nm LED source (IBH, UK, NanoLED-371L) and fluorescence signal at respective emission maximum was collected. Lifetime measurements were performed in duplicate and decay profiles were analyzed using IBH DAS6 analysis software. Fluorescence intensity decay profiles were found to be biexponential with χ^2 (goodness of fit) values very close to unity.

5. Quantum yield determination of emissive PNA base analog 5

The quantum yield of emissive PNA analog **5** in different solvents relative to cumarin 153 standard was determined using the following equation.[S4]

$\Phi_{F(x)} = (A_s/A_x)\ (F_x/F_s)\ (n_x/n_s)^2 \Phi_{F(s)}$

Where s is the standard, x is PNA analog (**5**), A is the absorbance at excitation wavelength, F is the area under the emission curve, n is the refractive index of the solvent, and Φ_F is the quantum yield. Quantum yield of cumarin 153 in acetonitrile is 0.56.[S5]

Quantum yield of PNA **10**: 0.22 ± 0.01
PNA **10** and poly(A) RNA ON **12** duplex (**10•12**): 0.29 ± 0.01
PNA-DNA duplex **10•11**: 0.17 ± 0.01

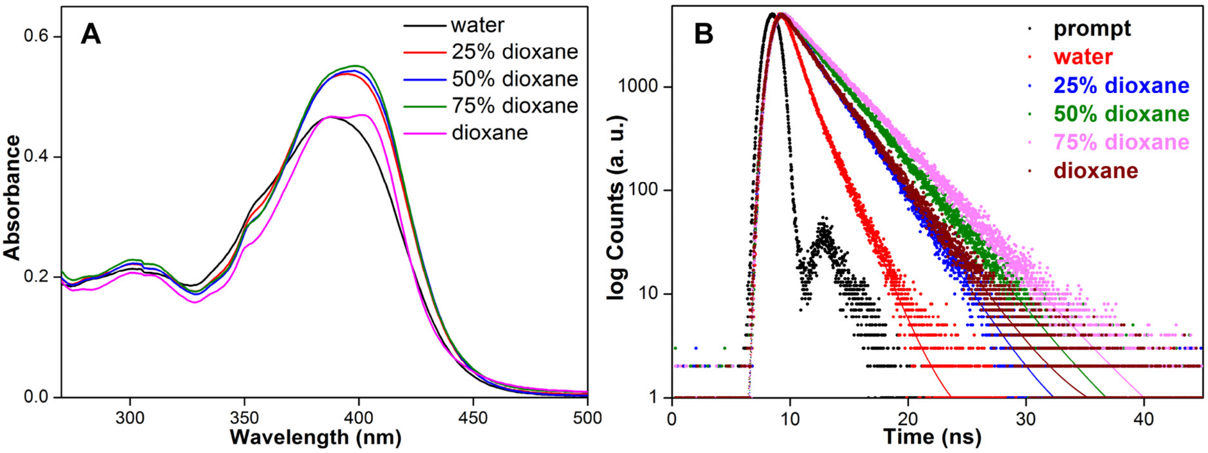

Figure S1. (**A**) Absorption spectra of PNA analog **5** (25.0 μM) in various solvents such as water, dioxane and their mixtures. All solutions contained 2.5% DMSO. (**B**) Excited-state decay profile (5.0 μM) of free PNA analog **5** in solvents of different polarity. Samples were excited using a 371 nm LED sources. The laser profile is shown in black (prompt). Curve fits are shown in solid lines. See Table 1 for details.

6. Solid-phase synthesis of control unmodified and naphthalimide-modified PNA oligomers

Model 15mer control unmodified (**6–9**) and fluorescently-modified (**6X–9X**) PNA oligomers were synthesized on MBHA resin using Boc-protected PNA monomers and Boc-protected fluorescent naphthalimide PNA acid **4** (Scheme S1) according to our reported protocol.[S6] Fluorescent poly(T) PNA probe **10** was synthesized by SPPS protocol on Rink amide resin using Fmoc-protected *aeg*-thymine and fluorescent naphthalimide-modified uracil PNA acid **f** (Scheme S2). The detailed protocol for the synthesis of poly(T) PNA probe **10** and unmodified poly(T) PNA **13** using Fmoc-chemistry is given below. To enhance aqueous solubility of the PNA oligomers, two lysine residues were attached at the C-terminus of all the PNA oligomers.

In a glass sintered flask, Rink-amide resin (250 mg, 0.65 mmol/g) was swelled in CH_2Cl_2 (8 mL) for 12 h. The solvent was then removed, and the resin was treated with 20% piperidine in DMF (6 mL) for 10 min to remove Fmoc-group from the resin. This step was repeated two more times. The resin was then washed sequentially with DMF (3 x 3 mL), CH_2Cl_2 (3 x 3 mL), and DMF (3 x 3 mL). The resin was dried under nitrogen flow for few minutes. The coupling reaction was performed in dry DMF (1.8 mL) with Fmoc-Lys(Boc)-OH (36 mg, 1.0 equiv. to obtain a loading of 0.35 mmol/g) in the presence of HOBt (1.0 equiv.), HBTU (1.0 equiv.) and DIPEA (1.0 equiv.) for 7–9 h at RT. The resin was further washed with DMF (3 x 3 mL), CH_2Cl_2 (3 x 3 mL), and DMF (3 x 3 mL). Next, remaining amino groups on the resin was capped with acetic anhydride (1.0 mL) in pyridine (1.0 mL) for 1 h at RT. This step was repeated two more times, and the resin was then washed with DMF (3 x 3 mL), CH_2Cl_2 (3 x 3 mL), and DMF (3 x 3 mL) and dried under nitrogen flow for few minutes. The resin was treated with 20% piperidine in DMF (5 mL) for 10 min to remove Fmoc-group. This step was repeated two more times, and the resin was washed with DMF (3 x 3 mL), CH_2Cl_2 (3 x 3 mL), and DMF (3 x 3 mL). Then coupling reaction was performed in dry DMF (1.8 mL) with Fmoc-Lys(Boc)-OH (3.0 equiv.) in the presence of HOBt (3.0 equiv.), HBTU (3.0 equiv.) and DIPEA (3.0 equiv.) for 7–9 h at RT. The resin was then washed with DMF (3 x 3 mL), CH_2Cl_2 (3 x 3 mL), and DMF (3 x 3 mL). The resin was dried under nitrogen flow for few minutes.

Above lysine loaded Rink-amide resin (25 mg, 0.35 mmol/g) was swelled in CH_2Cl_2 (2 mL) for 2 h in glass sintered flask. The solvent was removed and the resin was treated with 20% piperidine in DMF (1.0 mL) for 10 min to remove Fmoc-group as mentioned above. The resin was then washed with DMF (3 x 3 mL), CH_2Cl_2 (3 x 3 mL), and DMF (3 x 3 mL). The coupling reaction was performed in dry DMF (0.6 mL) with Fmoc-thymine PNA monomer (3.0 equiv.) in the presence of HOBt (3.0 equiv.), HBTU (3.0 equiv.) and DIPEA (3.0 equiv.) for 6 min at 65 °C using microwave peptide synthesizer (*Note*: coupling reaction for fluorescent PNA monomer **f** was carried out at RT for 7–9 h). The resin was washed again, and the coupling, Fmoc deprotection, and washing steps were repeated in the cycle as mentioned above to synthesize the desired PNA sequence. Also, a double-coupling reaction was performed for only fluorescently-modified PNA monomer **f** in the presence of HOBt (3.0 equiv.), HBTU (3.0 equiv.) and DIPEA (3.0 equiv.) for 7–9 h at RT.

Cleavage procedure: The dried resin (20 mg) was transferred to a glass vial and treated with H_2O (10 μL) and anisole (10 μL) in an ice bath for 10 min. TFA (380 μL) was then added to the above mixture and was stirred for 1.5 h at room temperature. The resin was filtered, and the filtrate was concentrated and precipitated as a white solid by adding cold diethyl ether (1 mL). The solvent was decanted, and the crude product was dissolved in autoclaved water and purified by RP-HPLC.

7. HPLC analysis of PNA oligomers

All control and fluorescently modified PNA oligomers were purified using a Luna C18 semi-preparative RP column (5 micron, 250 × 10 mm, Phenomenex) on an Agilent Technologies 1260 Infinity HPLC system. Conditions: 0–50% B in 25 min and 50–100% B in 15 min (Mobile phase A: 5% acetonitrile in H_2O containing 0.1% TFA. Mobile phase B: 50% acetonitrile in H_2O containing 0.1% TFA). Flow rate was 2 mL/min. The chromatogram of control PNA oligomers was recorded at 260 nm, and fluorescently modified PNA oligomers was recorded at 260 nm, and 400 nm. Concentration of the PNA oligomers was determined by using the molar extinction coefficients of nucleobases at 260 nm (ε_A = 13700, ε_T = 8600, ε_G = 11700, ε_C = 6600, ε_5 = 9680 $M^{-1}cm^{-1}$).[S7]

8. MALDI-TOF mass measurement of PNA oligomers

1 µL of a ~150 µM stock solution of PNA oligomer was combined with 2,5-dihydroxybenzoic acid (DHB) or α-cyano-4-hydroxycinnamic acid (CHCA) matrix. The samples were spotted on a plate and air dried before mass analysis.

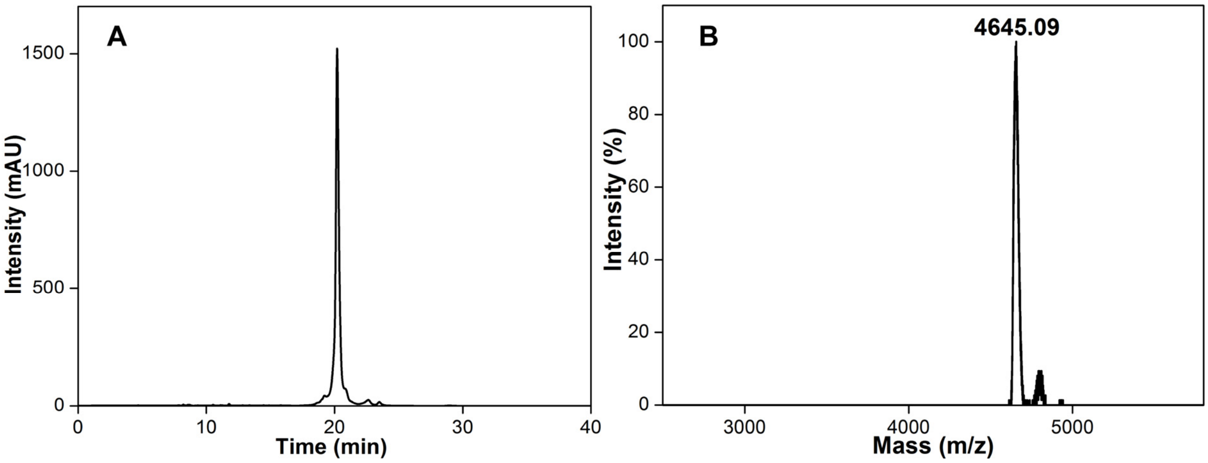

Figure S2. (**A**) Representative RP-HPLC chromatogram of fluorescently-modified PNA oligomer (e.g., **7X**). (**B**) Representative MALDI-TOF mass spectrum of fluorescently-modified PNA oligomer (e.g., **7X**). Calculated for $[M]^+$ = 4644.56 and observed = 4645.09.

Table S1. ε_{260} and MALDI-TOF mass analysis of PNA oligomers

PNA	Sequence[a]	ε_{260} ($M^{-1}cm^{-1}$)	Calculated mass	Observed mass
**6X**	CGATCAA**5**AACTAGCKK	15.89 x 10^4	4662.67 $[M]^+$	4663.84
**7X**	CGATCAT**5**TACTAGCKK	14.87 x 10^4	4644.56 $[M]^+$	4645.09
**8X**	CGATCAG**5**GACTAGCKK	15.49 x 10^4	4694.67 $[M]^+$	4697.30
**9X**	CGATCAC**5**CACTAGCKK	14.47 x 10^4	4614.54 $[M]^+$	4616.70
**10**	TTTTT**5**TTTTTTKK	10.43 x 10^4	3842.81 $[M+Na]^+$	3842.42
**13**	TTTTTTTTTTTTKK	10.32 x 10^4	3468.47 $[M]^+$	3468.43

[a]PNA sequences are written from N to C terminus.

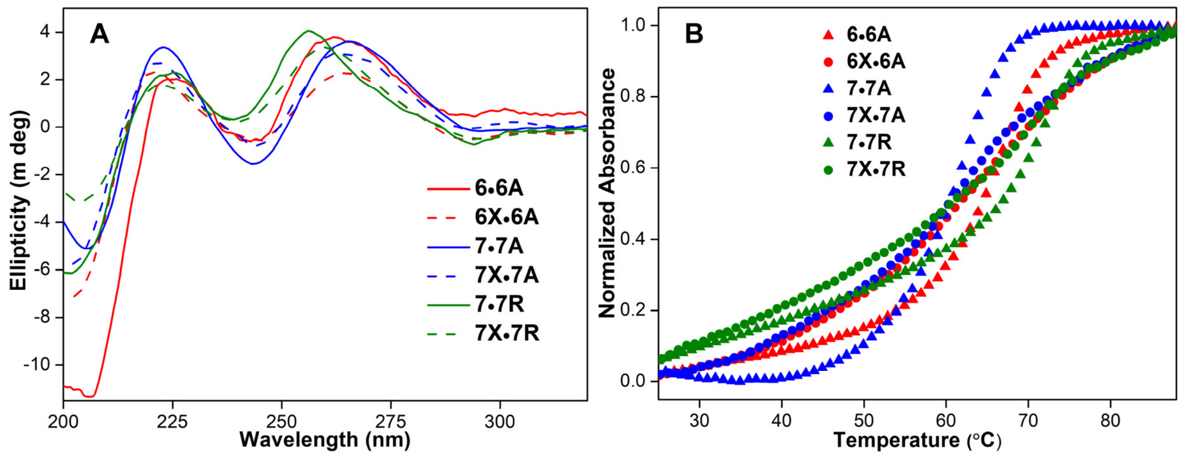

Figure S3. (**A**) CD spectra (5.0 µM) of control-unmodified (**6•6A**, **7•7A** and **7•7R**, solid line) and fluorescently-modified (**6X•6A**, **7X•7A** and **7X•7R**, dashed line) PNA-DNA and PNA-RNA ON duplexes in 10 mM phosphate buffer (pH 7.1, 100 mM NaCl, 0.1 mM EDTA). (**B**) UV-thermal melting profile (1.0 µM) of control-unmodified (**6•6A**, **7•7A**, and **7•7R**, filled triangle) and fluorescently-modified (**6X•6A**, **7X•7A** and **7X•7R**, filled circle) PNA-DNA and PNA-RNA ON duplexes in 10 mM phosphate buffer (pH 7.1, 100 mM NaCl, 0.1 mM EDTA). For T_m values see Table S2.

Table S2. T_m values of control unmodified and fluorescently modified PNA-ON duplexes.

Control unmodified duplex	T_m (°C)	Duplex made of **5**	T_m (°C)
**6•6A**	66.8 ± 0.4	**6X•6A**	61.8 ± 1.2
**7•7A**	62.3 ± 0.5	**7X•7A**	61.5 ± 0.8
**7•7T**	56.6 ± 1.0	**7X•7T**	55.0 ± 0.7
**7•7G**	52.7 ± 0.4	**7X•7G**	50.5 ± 0.4
**7•7C**	52.4 ± 0.7	**7X•7C**	53.2 ± 0.5
**7•7R**	71.8 ± 0.5	**7X•7R**	69.7 ± 0.9
**8•8A**	74.6 ± 1.1	**8X•8A**	63.2 ± 0.9
**9•9A**	70.1 ± 0.5	**9X•9A**	64.2 ± 1.3

Thermal melting experiments indicated that the modification had discernible destabilization effect on the duplex. However, the Lucifer-modified PNA oligomers formed stable hybrids with complementary ONs under the conditions used for fluorescence analysis.

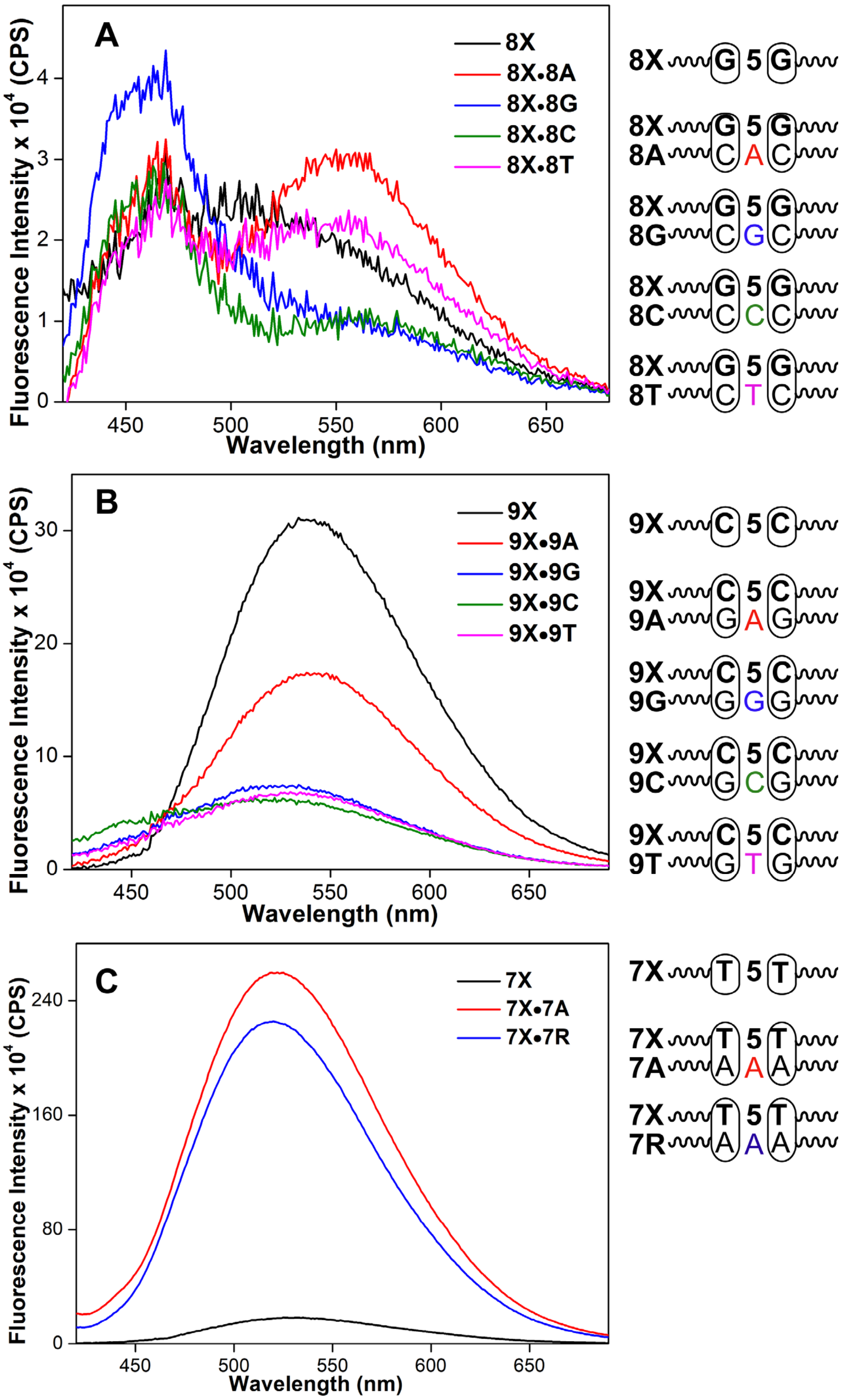

Figure S4. (**A**) Emission spectra (0.5 µM) of emissive PNA oligomer **8X** and PNA-DNA duplexes made of **8X**. (**B**) Emission spectra (0.5 µM) of emissive PNA oligomer **9X** and PNA-DNA duplexes made of **9X**. Excitation and emission slit widths were kept at 6 and 8 nm, respectively. PNA oligomers **8X** and **9X** and their duplexes were found to be very weakly fluorescent. (**C**) Emission spectra (0.5 µM) of emissive PNA oligomer **7X** and PNA-DNA/RNA duplex made of **7X**. Excitation and emission slit widths were kept at 6 and 7 nm, respectively. All samples were prepared in 10 mM phosphate buffer (pH 7.1, 100 mM NaCl, 0.1 mM EDTA) and excited at 400 nm.

A **RNA 14 with 3'-poly(A) tail:**

5'GGACCGAAAUUAAUACGACUCACUAUAGGGGUCUAUAUAAGCAGAGCUGGUUUAGU
GAACCGUCAGAUCCGCUAGCGCUACCGGACUCAGAUCUCGAGCUCAAGCUUCGAAU
UCUGCAGUCGACGGUACCGCGGGCCCGGGAUCCACCGGUCGCCACCAUGGUGAGCA
AGGGCGAGGAGCUGUUCACCGGGGUGGUGCCCAUCCUGGUCGAGCUGGACGGCGA
CGUAAACGGCCACAAGUUCAGCGUGUCCGGCGAGGGCGAGGGCGAUGCCACCUACG
GCAAGCUGACCCUGAAGUUCAUCUGCACCACCGGCAAGCUGCCCGUGCCCUGGCCC
ACCCUCGUGACCACCCUGACCUACGGCGUGCAGUGCUUCAGCCGCUACCCCGACCA
CAUGAAGCAGCACGACUUCUUCAAGUCCGCCAUGCCCGAAGGCUACGUCCAGGAGC
GCACCAUCUUCUUCAAGGACGACGGCAACUACAAGACCCGCGCCGAGGUGAAGUUC
GAGGGCGACACCCUGGUGAACCGCAUCGAGCUGAAGGGCAUCGACUUCAAGGAGGA
CGGCAACAUCCUGGGGCACAAGCUGGAGUACAACUACAACAGCCACAACGUCUAUAU
CAUGGCCGACAAGCAGAAGAACGGCAUCAAGGUGAACUUCAAGAUCCGCCACAACAU
CGAGGACGGCAGCGUGCAGCUCGCCGACCACUACCAGCAGAACACCCCCAUCGGCG
ACGGCCCCGUGCUGCUGCCCGACAACCACUACCUGAGCACCCAGUCCGCCCUGAGC
AAAGACCCCAACGAGAAGCGCGAUCACAUGGUCCUGCUGGAGUUCGUGACCGCCGC
CGGGAUCACUCUCGGCAUGGACGAGCUGUACAAGUAAAGCGGCCGCGACUCUAGAU
CAUAAUCAGCCAUACCACAUUUGUAGAGGUUUUACUUGCUUUAAAAAACCUCCCACA
CCUCCCCCUGAACCUGAAAAAAAAAAAAAAAAAAAAAAAAAAAAAAAAAAA 3'

RNA 15 without 3'-poly(A) tail:

5'GGACCGAAAUUAAUACGACUCACUAUAGGGGUCUAUAUAAGCAGAGCUGGUUUAGU
GAACCGUCAGAUCCGCUAGCGCUACCGGACUCAGAUCUCGAGCUCAAGCUUCGAAU
UCUGCAGUCGACGGUACCGCGGGCCCGGGAUCCACCGGUCGCCACCAUGGUGAGCA
AGGGCGAGGAGCUGUUCACCGGGGUGGUGCCCAUCCUGGUCGAGCUGGACGGCGA
CGUAAACGGCCACAAGUUCAGCGUGUCCGGCGAGGGCGAGGGCGAUGCCACCUACG
GCAAGCUGACCCUGAAGUUCAUCUGCACCACCGGCAAGCUGCCCGUGCCCUGGCCC
ACCCUCGUGACCACCCUGACCUACGGCGUGCAGUGCUUCAGCCGCUACCCCGACCA
CAUGAAGCAGCACGACUUCUUCAAGUCCGCCAUGCCCGAAGGCUACGUCCAGGAGC
GCACCAUCUUCUUCAAGGACGACGGCAACUACAAGACCCGCGCCGAGGUGAAGUUC
GAGGGCGACACCCUGGUGAACCGCAUCGAGCUGAAGGGCAUCGACUUCAAGGAGGA
CGGCAACAUCCUGGGGCACAAGCUGGAGUACAACUACAACAGCCACAACGUCUAUAU
CAUGGCCGACAAGCAGAAGAACGGCAUCAAGGUGAACUUCAAGAUCCGCCACAACAU
CGAGGACGGCAGCGUGCAGCUCGCCGACCACUACCAGCAGAACACCCCCAUCGGCG
ACGGCCCCGUGCUGCUGCCCGACAACCACUACCUGAGCACCCAGUCCGCCCUGAGC
AAAGACCCCAACGAGAAGCGCGAUCACAUGGUCCUGCUGGAGUUCGUGACCGCCGC
CGGGAUCACUCUCGGCAUGGACGAGCUGUACAAGUAAAGCGGCCGCGACUCUAGAU
CAUAAUCAGCCAUACCACAUUUGUAGAGGUUUUACUUGCUUUAAAAAACCUCCCACA
CCUCCCCCUGAACCUG 3'

B

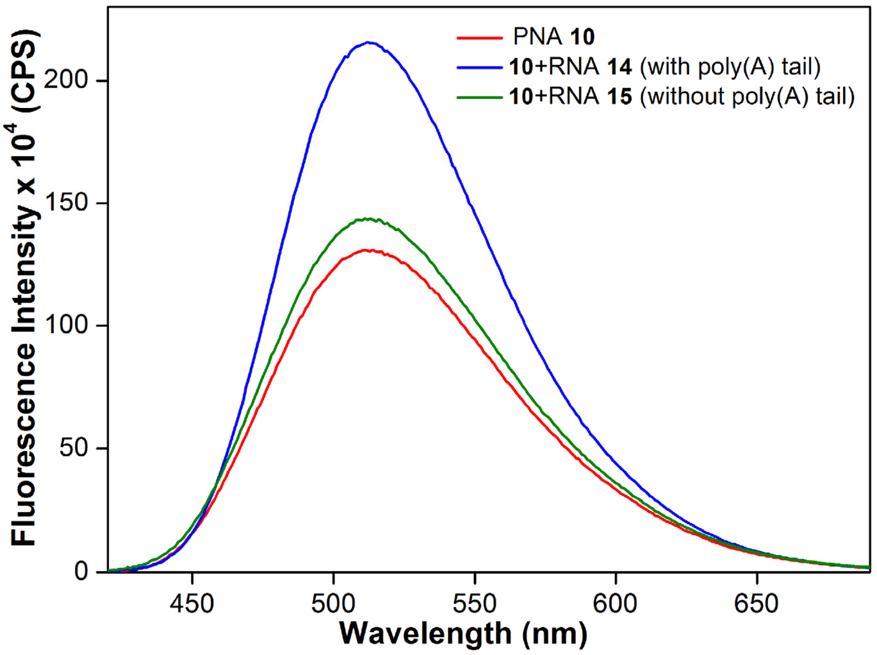

Figure S5. (**A**) Sequence of the longer RNA transcript **14** containing a 3′ poly(A) tail (35 adenosine residues, shown in red color) and control RNA transcript **15** without 3′ poly(A) tail. (**B**) Fluorescence spectrum of PNA **10** (red line) and PNA **10** incubated with RNA transcripts **14** (blue line) and **15** (green line). See experimental section for details.

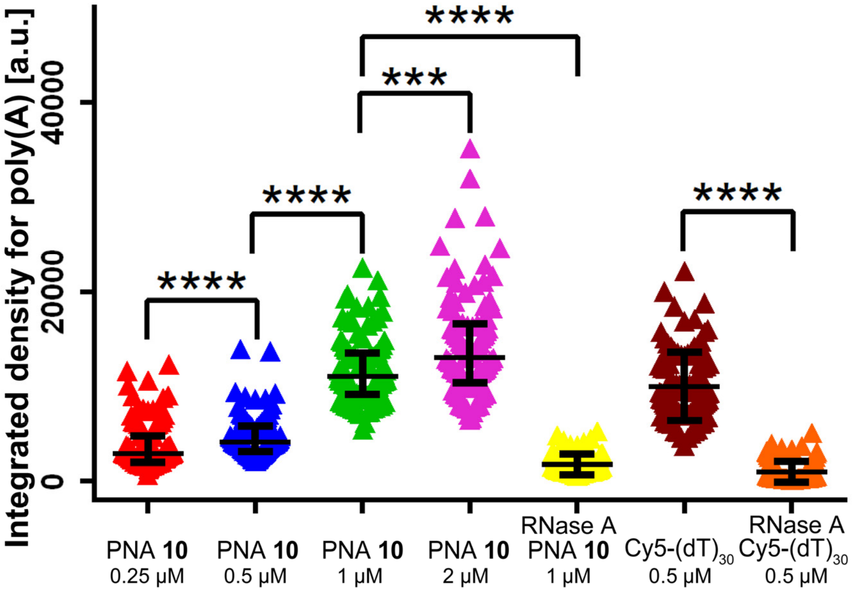

Figure S6. The distribution of poly(A) RNAs in DLD1 cells was determined by quantifying the integrated density in cells (nucleus and cytoplasm). Scatter plots showing normalized integrated density for poly(A) signals upon binding of PNA probe **10** at 0.25 µM (n = 113), 0.5 µM (n = 97), 1.0 µM (n = 104) and 2.0 µM (n = 101) and Cy5-(dT)$_{30}$ (0.5 µM, n = 106). DLD1 cells treated with RNase A and hybridized with PNA **10** (1.0 µM, n = 104) and Cy5-(dT)$_{30}$ (0.5 µM, 92) were used as controls. Normalized integrated densities were calculated using Image J software and Scatter plots were obtained using GraphPad Prism software. Horizontal line represents median, p values obtained from Mann–Whitney U test, n is total number of cells used for quantification (****$p < 0.0001$, ***$p < 0.001$).

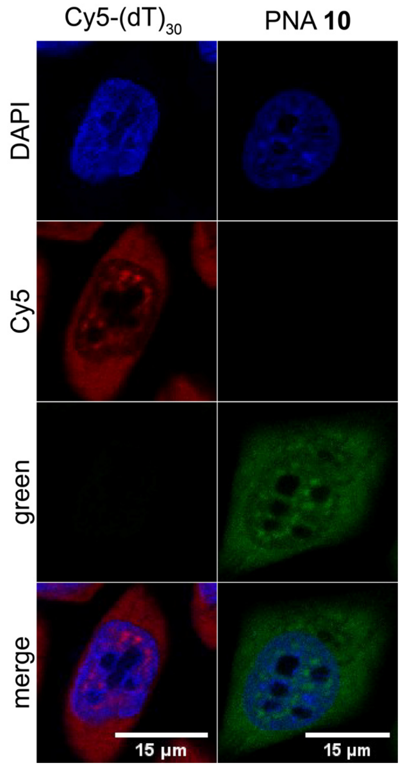

Figure S7. Magnified confocal images of poly(A) RNAs-stained DLD1 cells using Cy5-(dT)$_{30}$ (0.5 µM, red) and poly(T) PNA **10** (1.0 µM, green). Cultured DLD1 cells were fixed, permeabilized and hybridized with Cy5-(dT)$_{30}$ (0.5 µM) or PNA **10** (1.0 µM).

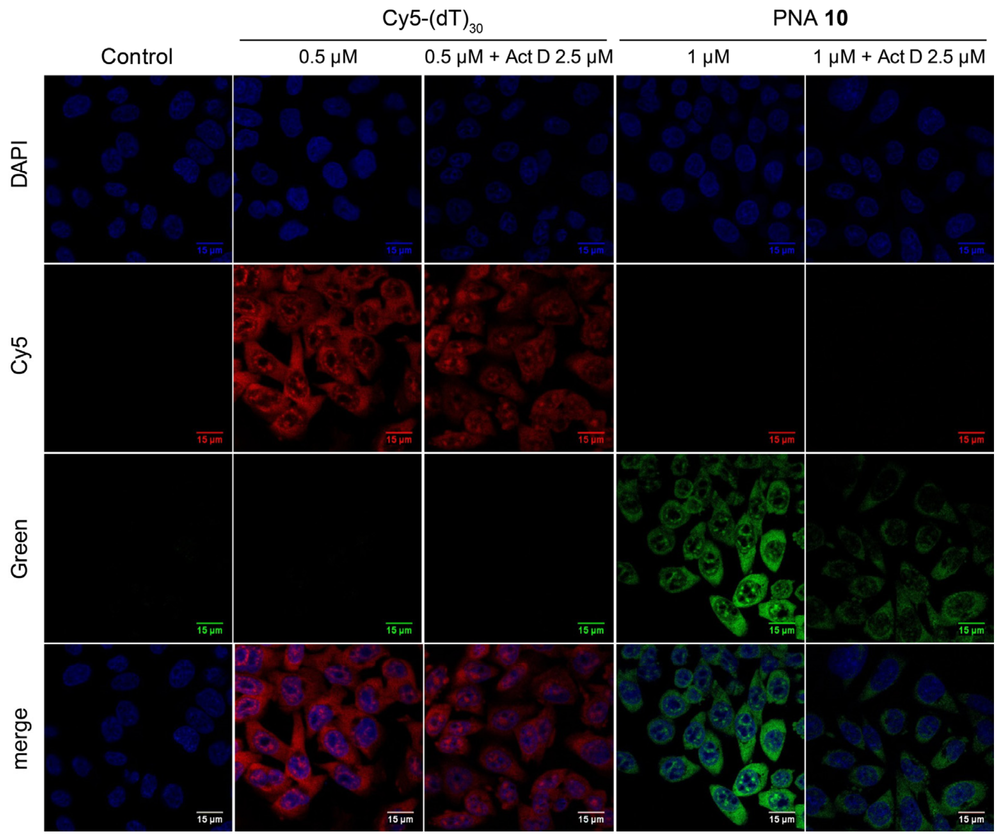

Figure S8. Effect of polymerase inhibitor on the poly(A) RNA staining by poly(T) PNA **10** and Cy5-$(dT)_{30}$. DLD1 cells in culture were treated with actinomycin D (Act D, 2.5 μM) and then subjected to hybridization with Cy5-$(dT)_{30}$ (0.5 μM) or PNA **10** (1.0 μM). Actinomycin D treatment displayed a noticeable reduction in fluorescence signal in both green as well as red channel. Cells were grown on the coverslips in RPMI1640 medium (300 μL) containing actinomycin D (2.5 μM) for 3 h and subjected to hybridization. See experimental section for more details.

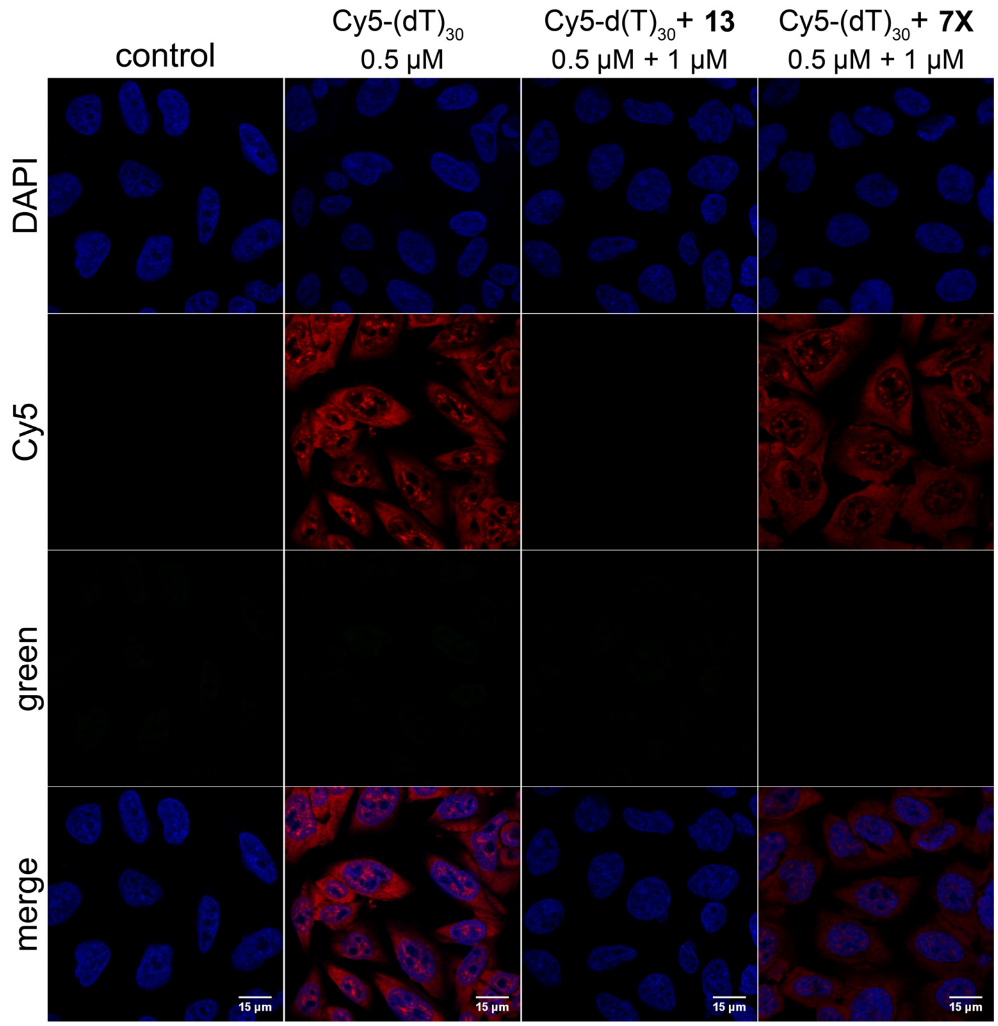

Figure S9. Competition assay between Cy5-$(dT)_{30}$ and unmodified poly$(T)_{12}$ PNA **13**, and Cy5-$(dT)_{30}$ and fluorescent PNA oligomer (**7X**) of a random sequence. Binding of non-fluorescent poly(T) PNA **13** to poly(A) RNAs abolished the signal from Cy5-$(dT)_{30}$ (red, column 3). However, a random sequence PNA **7X** did not affect the staining ability of Cy5-$(dT)_{30}$ (column 4).

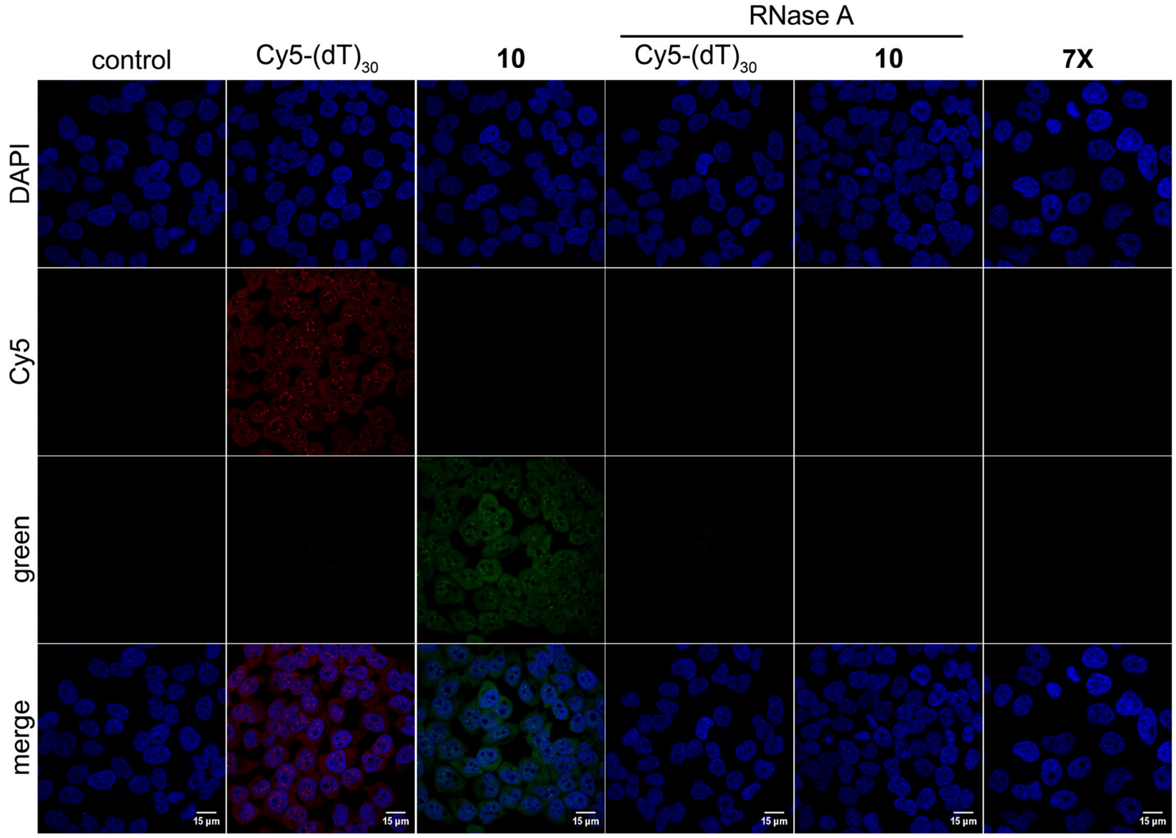

Figure S10. Column 2 and 3: Imaging cellular poly(A) RNAs using DNA ON Cy5-$(dT)_{30}$ and PNA probe **10** in HeLa cells. Cultured HeLa cells were fixed, permeabilized and hybridized with Cy5-$(dT)_{30}$ (0.5 μM, red) or PNA **10** (1.0 μM, green).

Column 4 and 5: HeLa cells were treated with RNase A and then subjected to hybridization with Cy5-$(dT)_{30}$ (0.5 μM) or PNA **10** (1.0 μM). RNase treatment almost completely eliminated the fluorescence signal from the cells incubated with DNA and PNA probes.

Column 6: A random sequence of naphthalimide-modified PNA oligomer **7X** (1.0 μM) did not stain poly(A) RNAs.

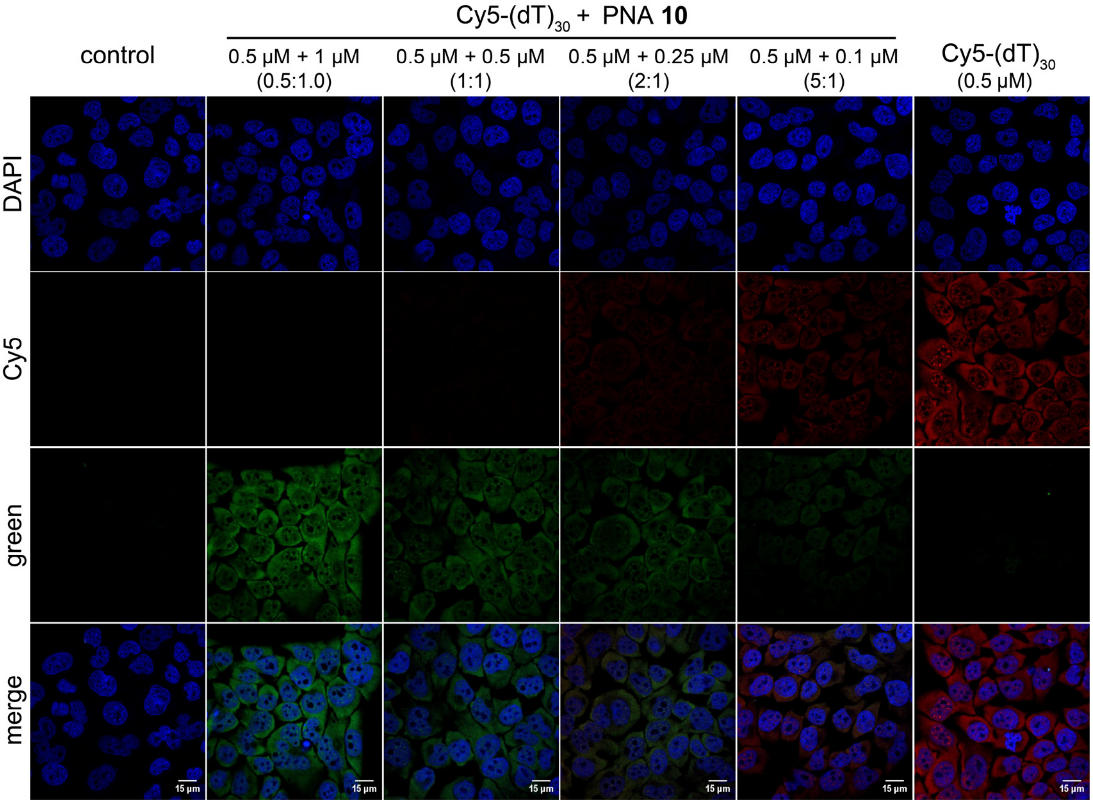

Figure S11. Competition assay indicates that PNA probe **10** binds to poly(A) RNAs of HeLa cells with higher affinity as compared to Cy5-(dT)$_{30}$. Cultured HeLa cells were fixed, permeabilized and hybridized with increasing molar ratios of Cy5-(dT)$_{30}$ to PNA **10**.

9. Binding assay: binding of poly(T) PNA 10 and Cy5-(dT)$_{30}$ to poly(A) RNA ON 12

Steady-state fluorescence: A series of solution of PNA **10** (0.5 µM) containing increasing concentrations of poly(A) RNA ON **12** (50 nM to 1 µM) was prepared in 10 mM phosphate buffer (pH 7.1, 100 mM NaCl, 0.1 mM EDTA) by heating at 90 ºC for 3 min. All samples were slowly cooled to RT and kept in an ice bath for ~1 h prior to fluorescence analysis. The samples were excited at 400 nm and changes in fluorescence intensity were recorded at 515 nm. The excitation and emission slit widths were kept at 3 nm and 4 nm, respectively. All fluorescence experiments were performed in triplicate in a micro fluorescence cuvette (Hellma, path length 1.0 cm) on a Fluoromax-4. Dissociation constant K_d was determined as explained below.

K_d determination: Normalized fluorescence intensity (F_N) versus log of poly(A) RNA **12** concentration plots were fitted using Hill equation (OriginPro 8.5.1) to determine the apparent binding constant K_d for the binding of **12** to PNA **10**.[S8]

$$F_N = \frac{F_i - F_s}{F_0 - F_s}$$

F_i is the fluorescence intensity at each titration point. F_0 and F_s are the fluorescence intensity in the absence of poly(A) RNA **12** and at saturation, respectively. L is concentration of ON **12**. n is the Hill coefficient or degree of cooperativity associated with the binding.

$$F_N = F_0 + (F_s - F_0)\left(\frac{[L]^n}{[K_d]^n + [L]^n}\right)$$

Gel shift assay: A series of solution of Cy5-$(dT)_{30}$ (1.0 µM) containing increasing concentrations of poly(A) RNA ON **12** (100 nM to 5 µM) was prepared as mentioned above. The volume of samples was 10 µL. To the samples was added 10 µL of loading buffer (10 mM Tris-HCl, pH 7.2 and 10% glycerol). The samples were loaded onto a 15% non-denaturing polyacrylamide gel containing 100 mM NaCl. A peristaltic pump was used to maintain the ionic conditions in both the chambers. The gel electrophoresis was performed at ~4 °C with a constant power supply of 14 W for ~10 h. The bands were visualized by using Typhoon-TRIO+ imager by illuminating the gel using fluorescence of Cy5. The bands were quantified using GeneTools image analysis software (Syngene) and K_d was determined as explained below.

K_d determination: Fraction bound (F_N) versus log of poly(A) RNA **12** concentration plots were fitted using Hill equation (OriginPro 8.5.1) to determine the apparent binding constant K_d for the binding of **12** to Cy5-$(dT)_{30}$.[S8]

$$F_N = F_0 - F_i$$

F_0 and F_i are band intensity in the absence of RNA ON **12** and at each titration point, respectively. Normalized F_N was used in the plot. F_s is band intensity at saturated amount of RNA ON **12**. L is concentration of ON **12**. n is the Hill coefficient or degree of cooperativity associated with the binding.

$$F_N = F_0 + (F_s - F_0)\left(\frac{[L]^n}{[K_d]^n + [L]^n}\right)$$

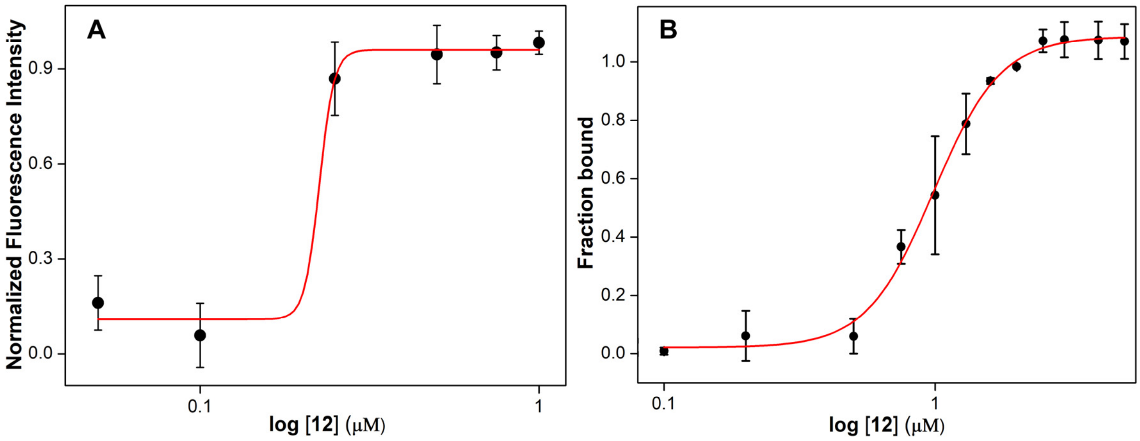

Figure S12. Curve fits for the binding of poly(T) PNA **10** or Cy5-$(dT)_{30}$ to poly(A) RNA ON **12**. (**A**) Normalized fluorescence intensity at λ_{em} (515 nm) is plotted against log [**12**] for poly(T) PNA **10**. (**B**) Fraction bound for binding of Cy5-$(dT)_{30}$ to poly(A) RNA ON **12** is plotted against log [**12**]. See section 9 for details.

10. NMR Spectra

^{1}H-NMR of compound **3** in $CDCl_3$

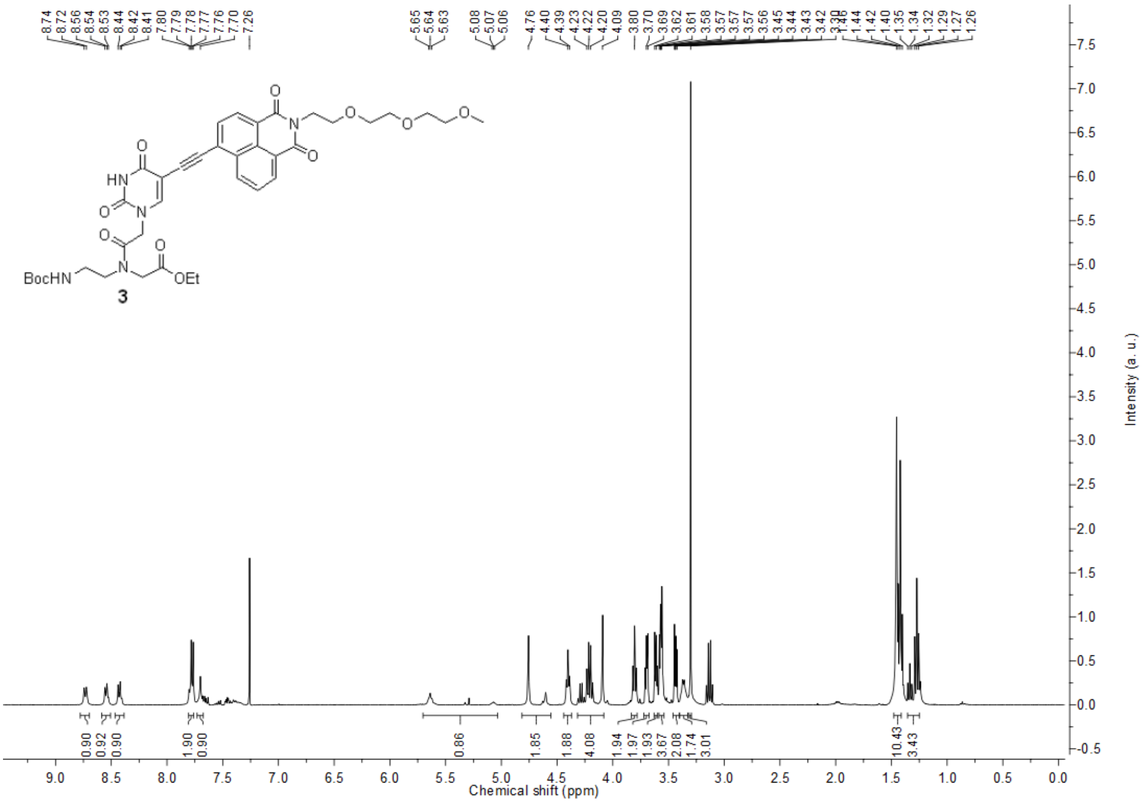

^{13}C-NMR of compound **3** in $CDCl_3$

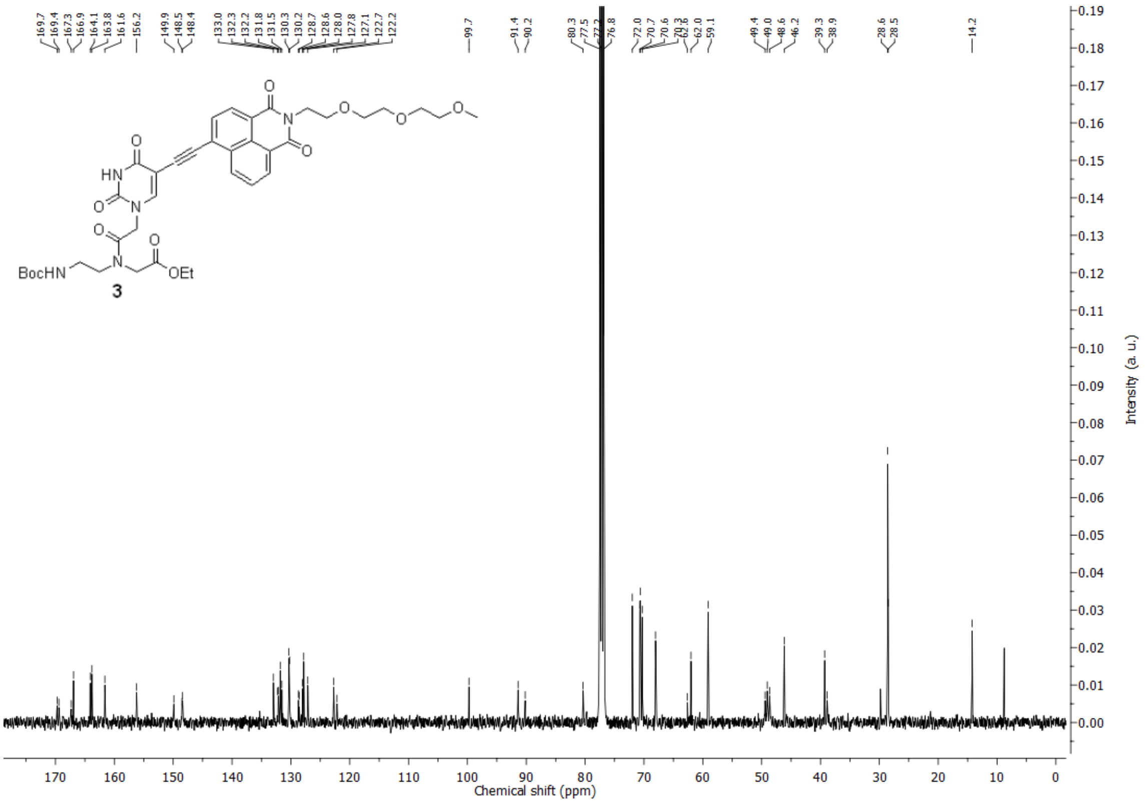

^{1}H-NMR of compound **4** in d_6-DMSO

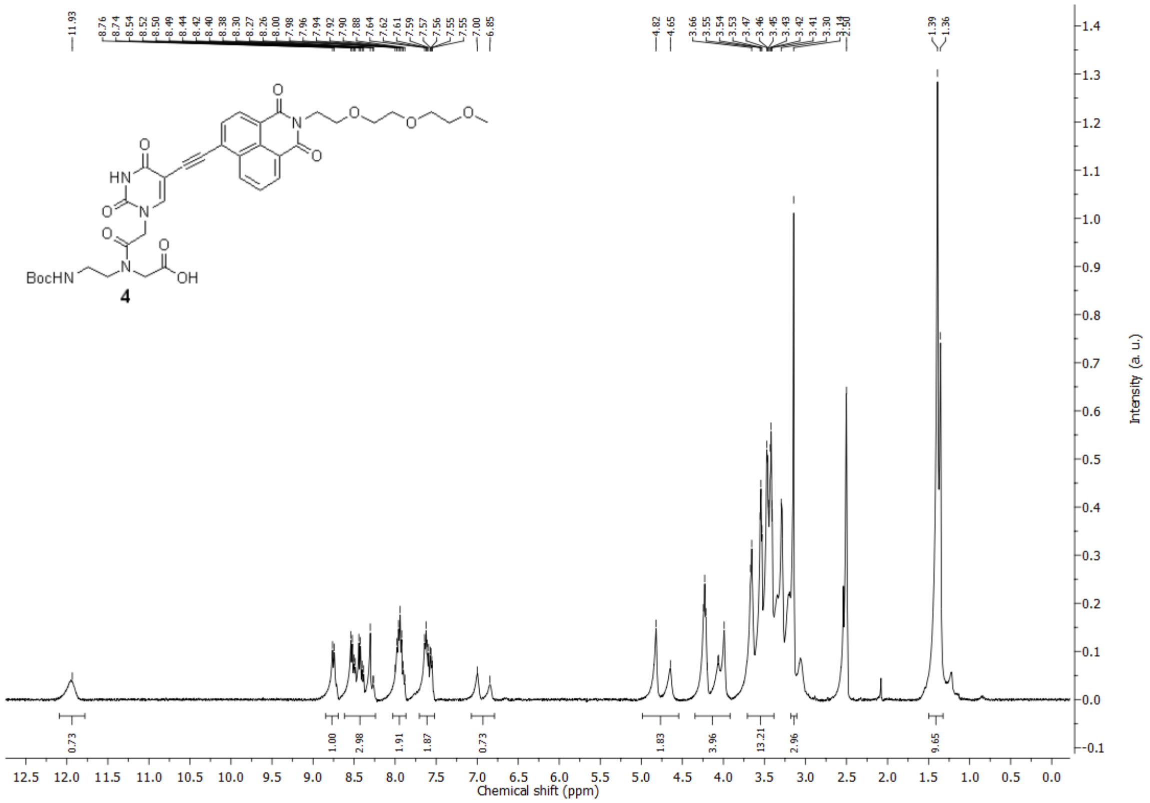

^{13}C-NMR of compound **4** in d_6-DMSO

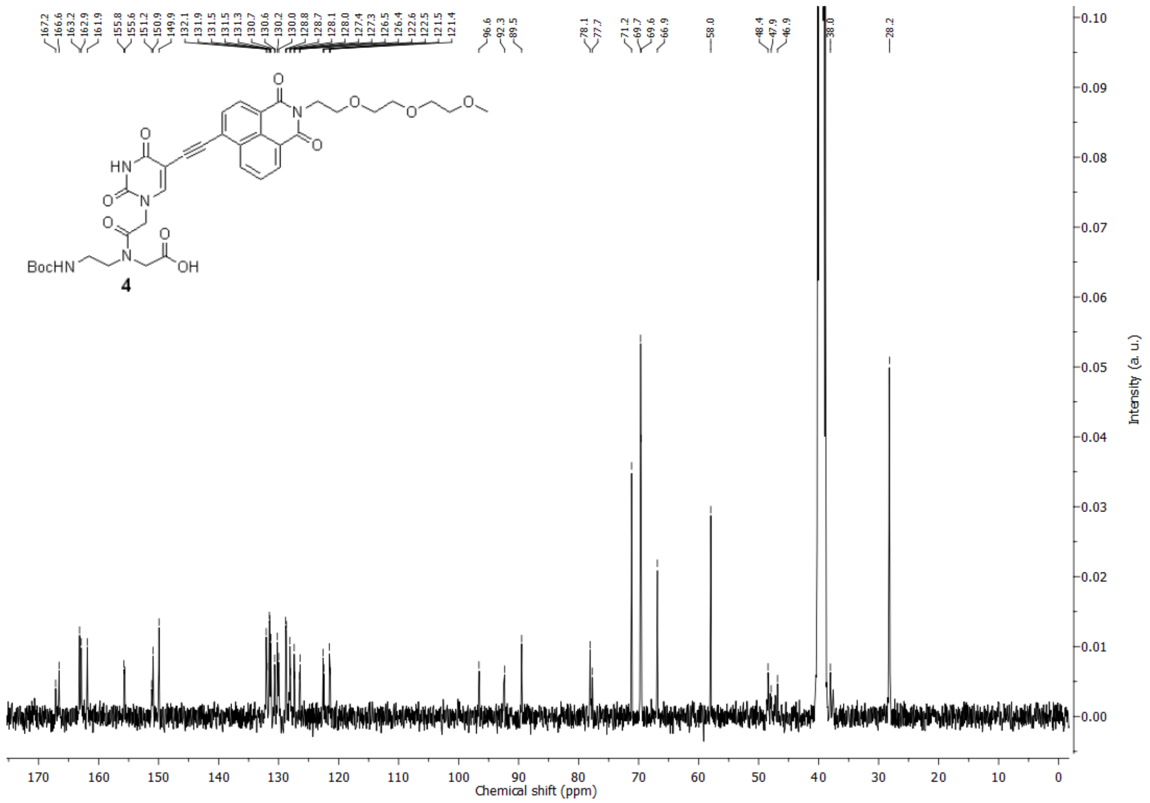

^{1}H-NMR of compound **5** in d_6-DMSO

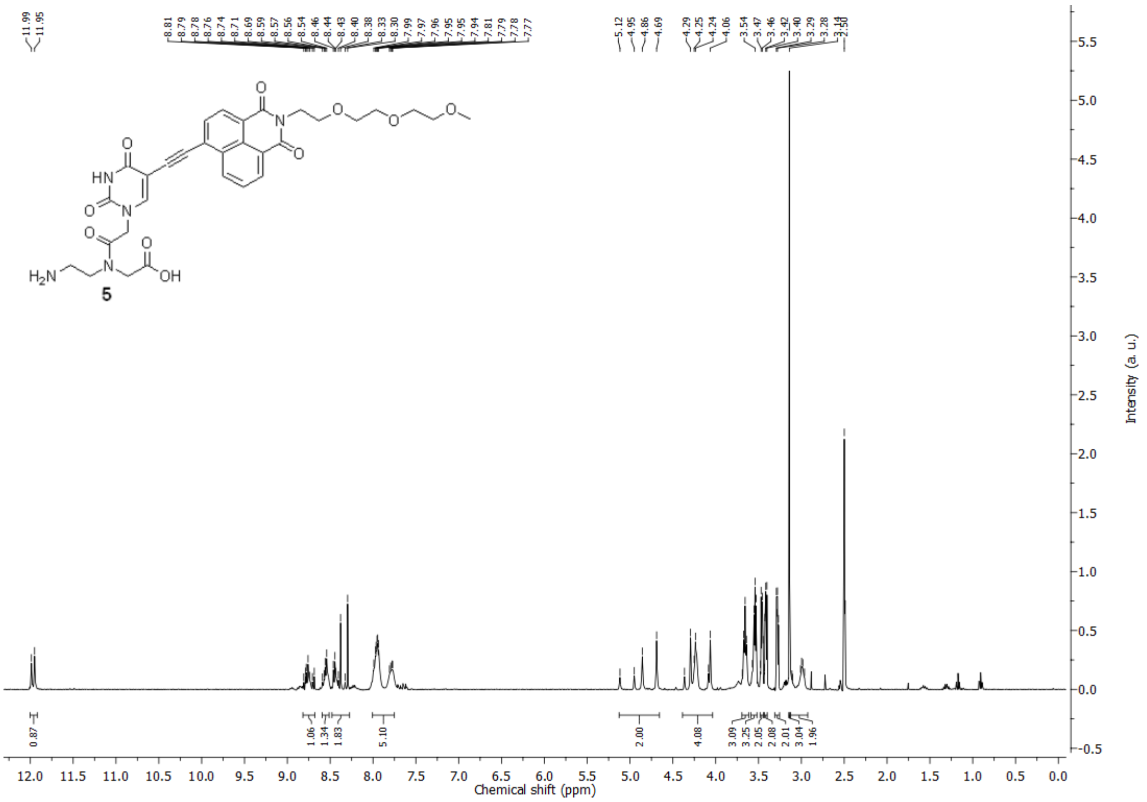

^{13}C-NMR of compound **5** in d_6-DMSO

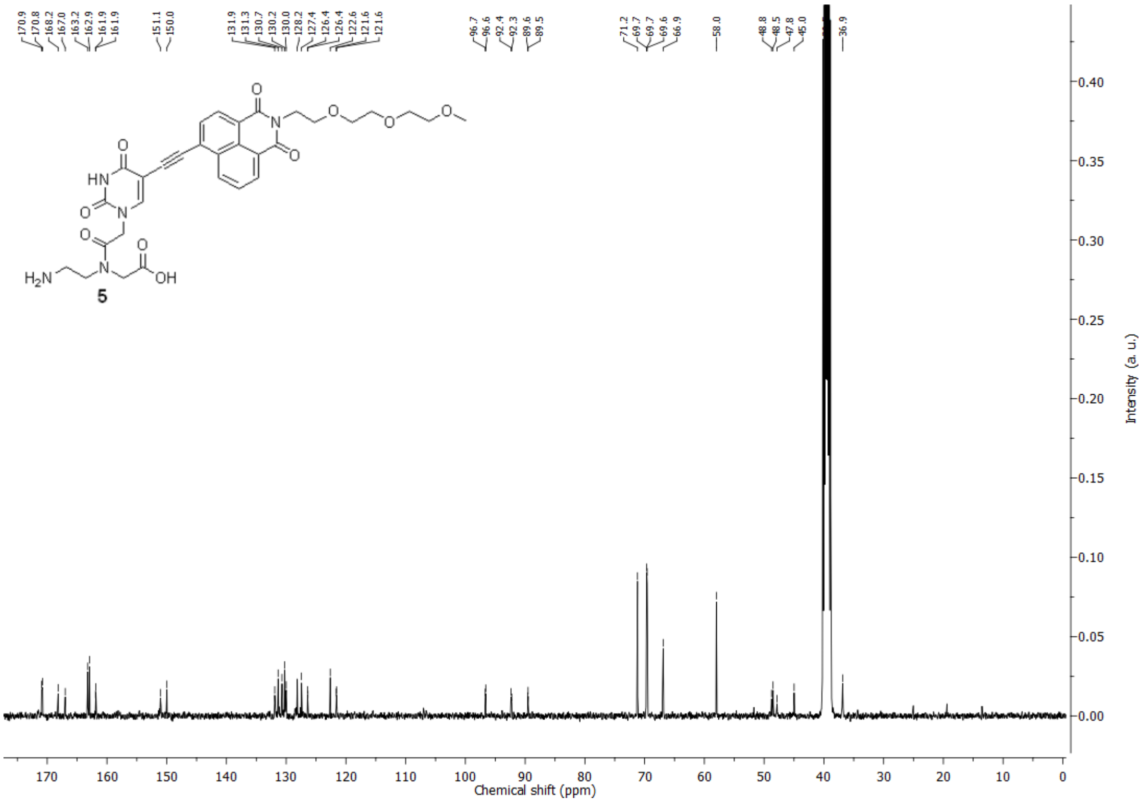

^{1}H-NMR of compound **b** in d_6-DMSO

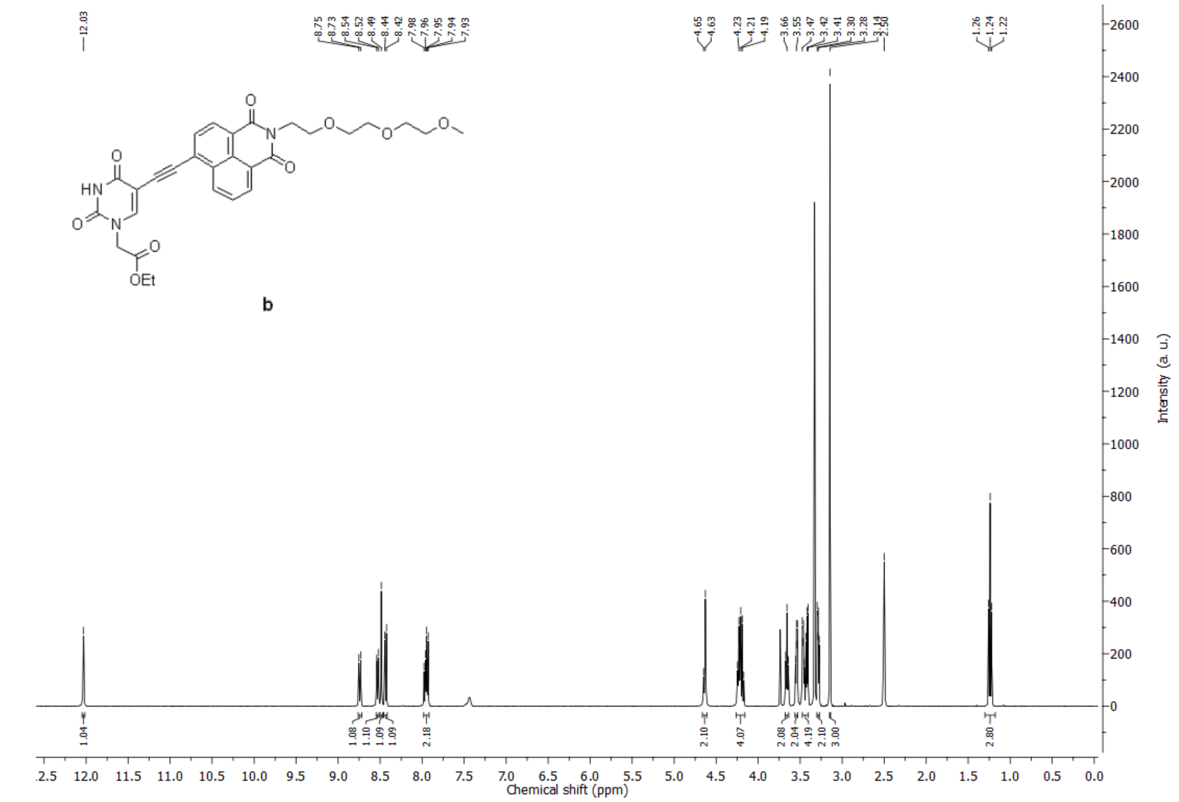

^{13}C-NMR of compound **b** in d_6-DMSO

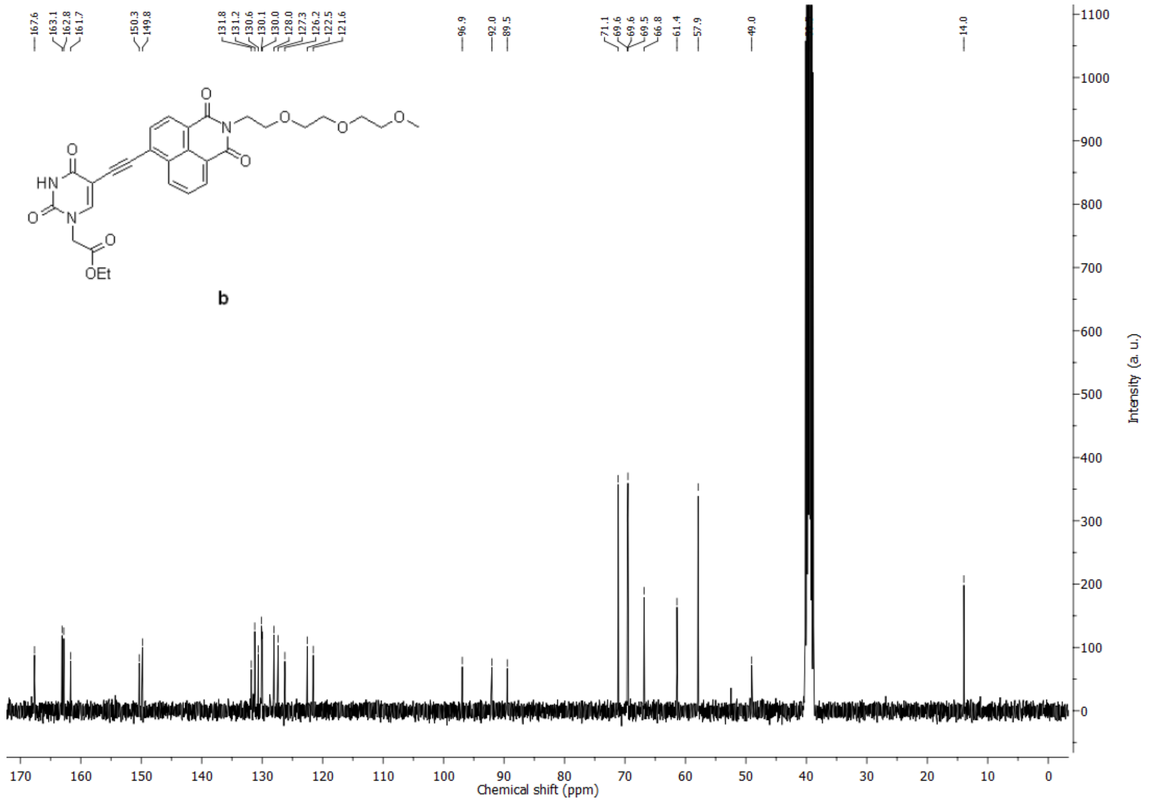

^{1}H-NMR of compound **c** in *d_6*-DMSO

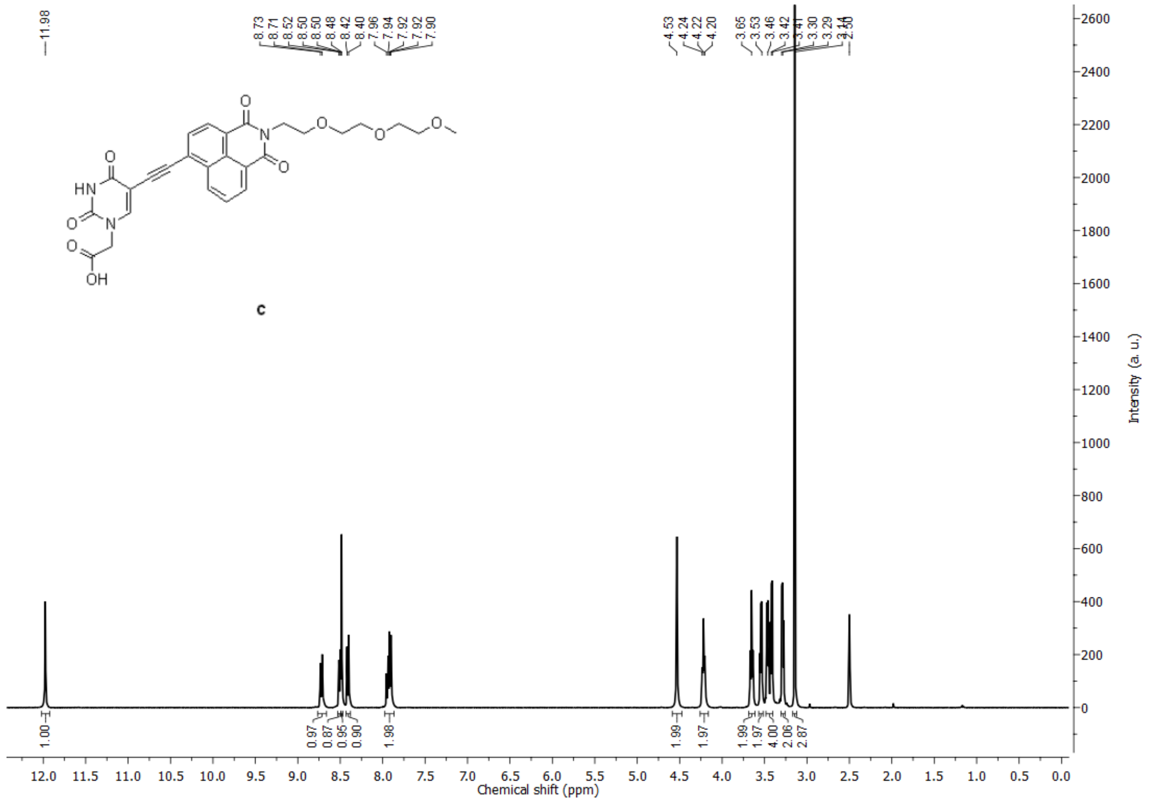

^{13}C-NMR of compound **c** in *d_6*-DMSO

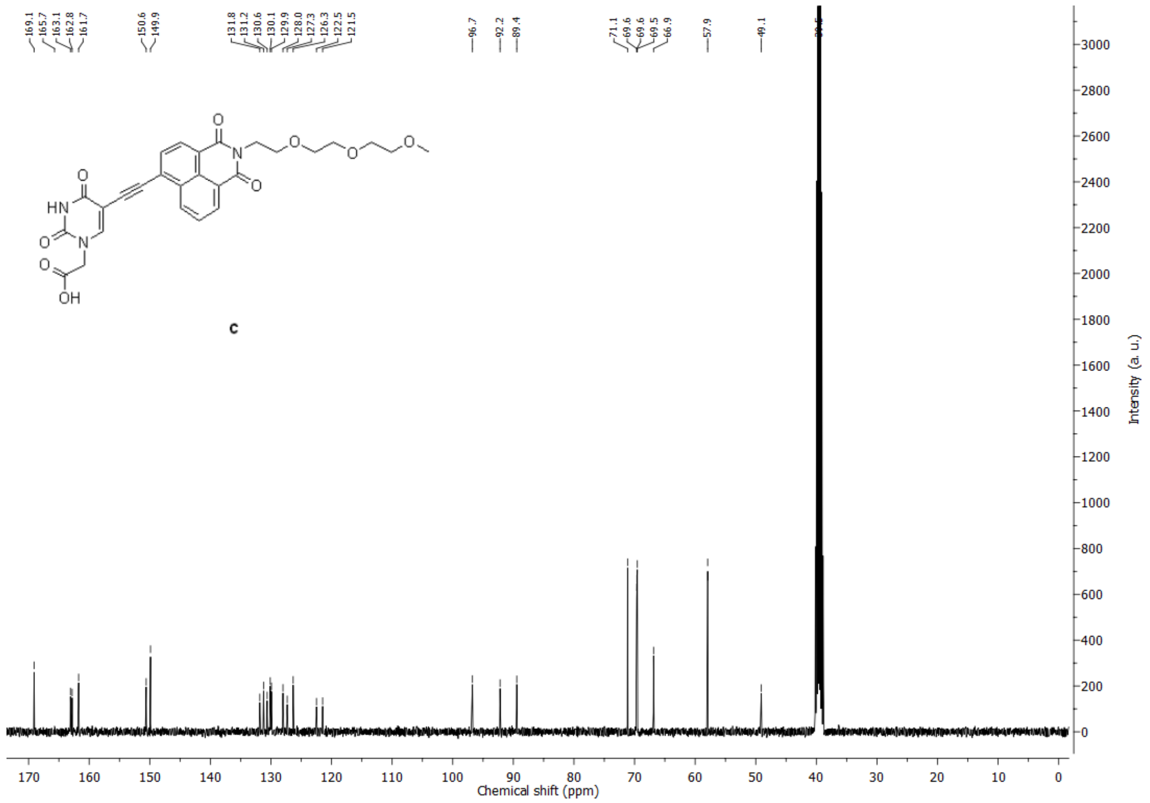

^{1}H-NMR of compound **e** in d_6-DMSO. Trace amount of CH_2Cl_2 was present (5.76 ppm)

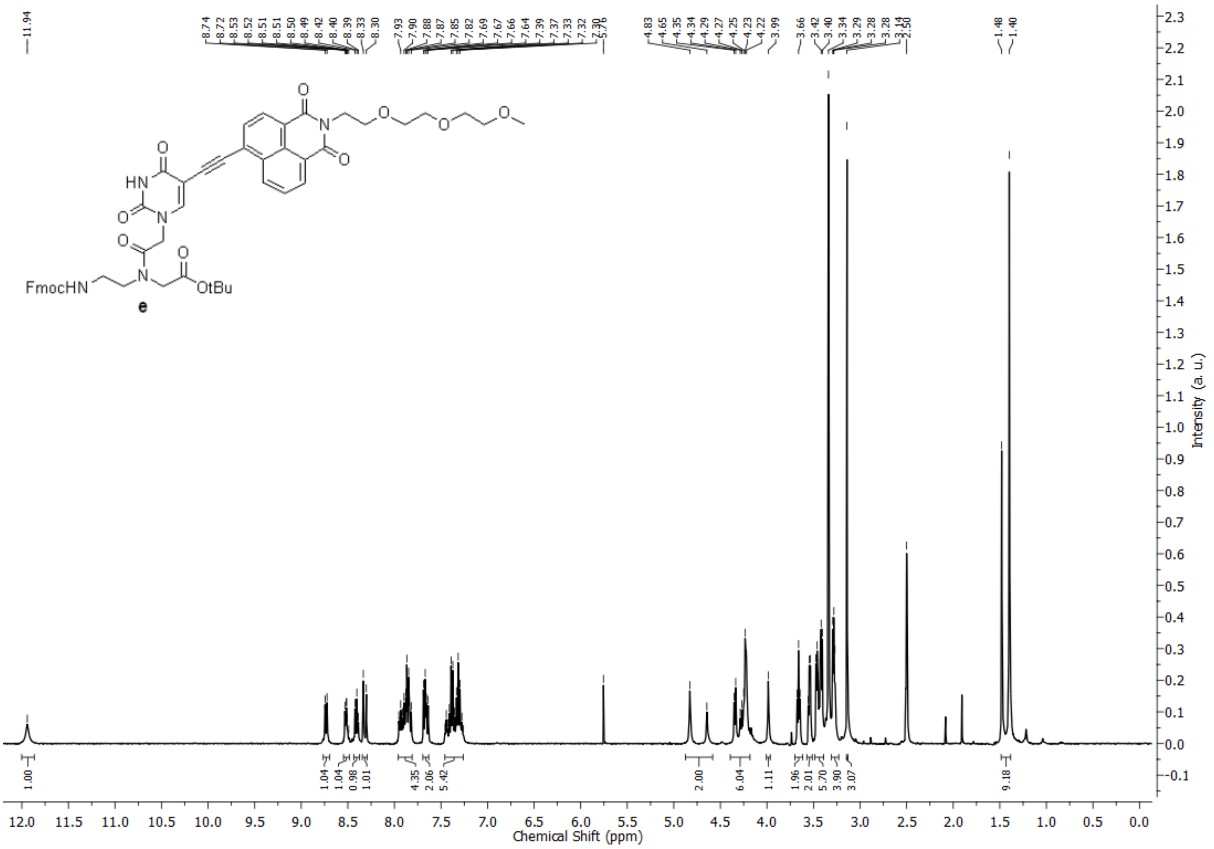

^{13}C-NMR of compound **e** in d_6-DMSO

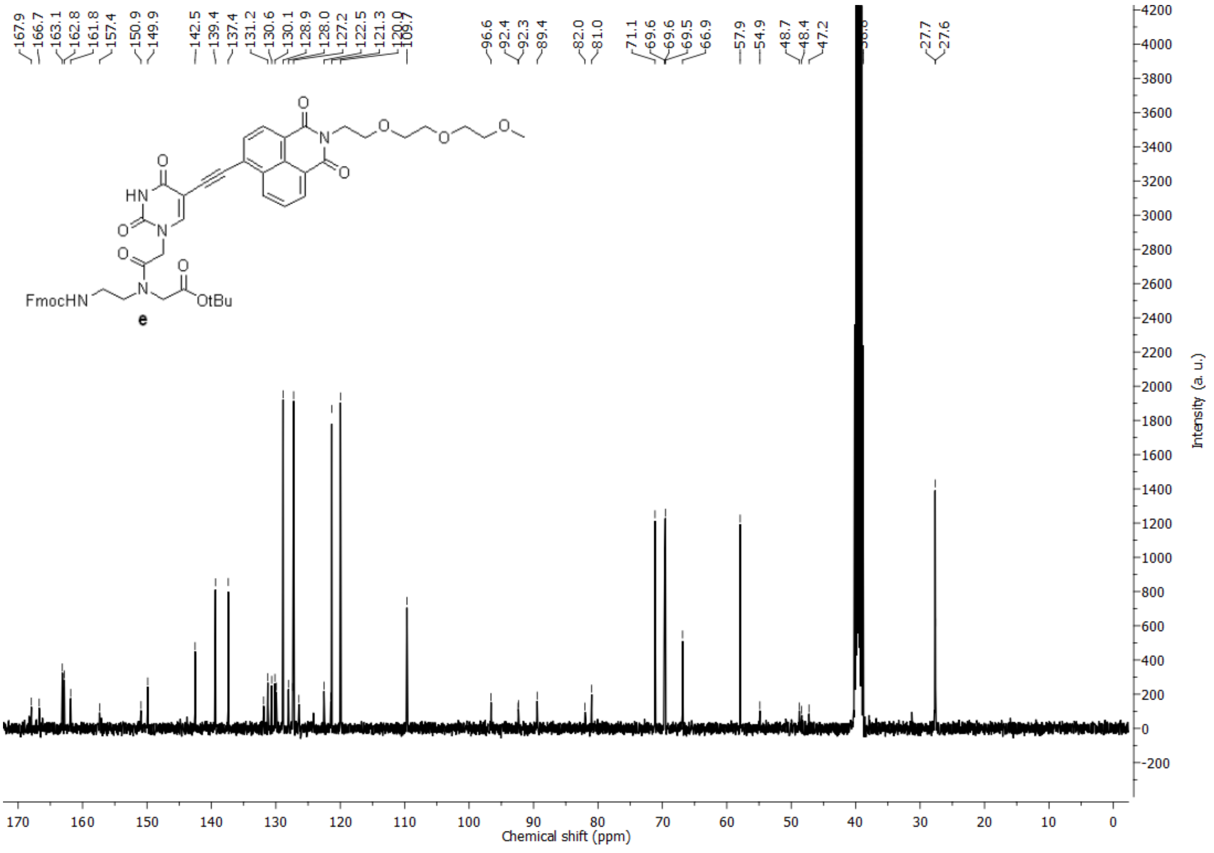

^{1}H-NMR of compound **f** in d_6-DMSO

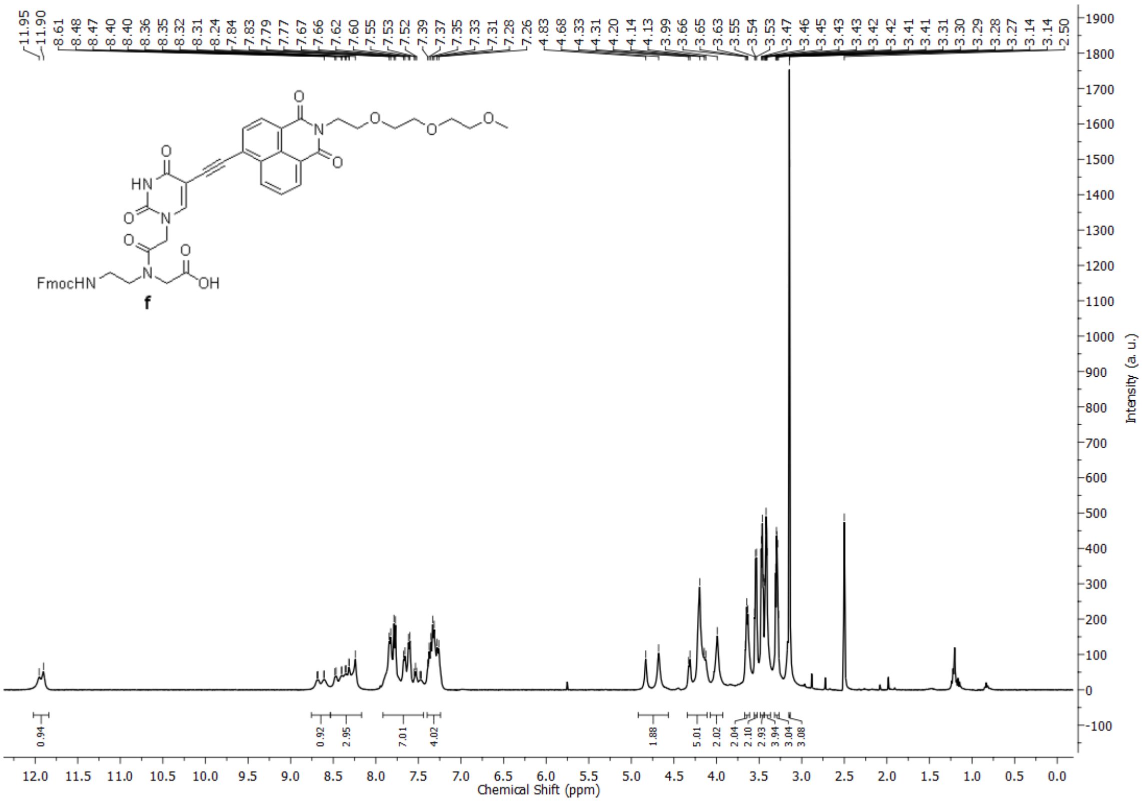

^{13}C-NMR of compound **f** in d_6-DMSO

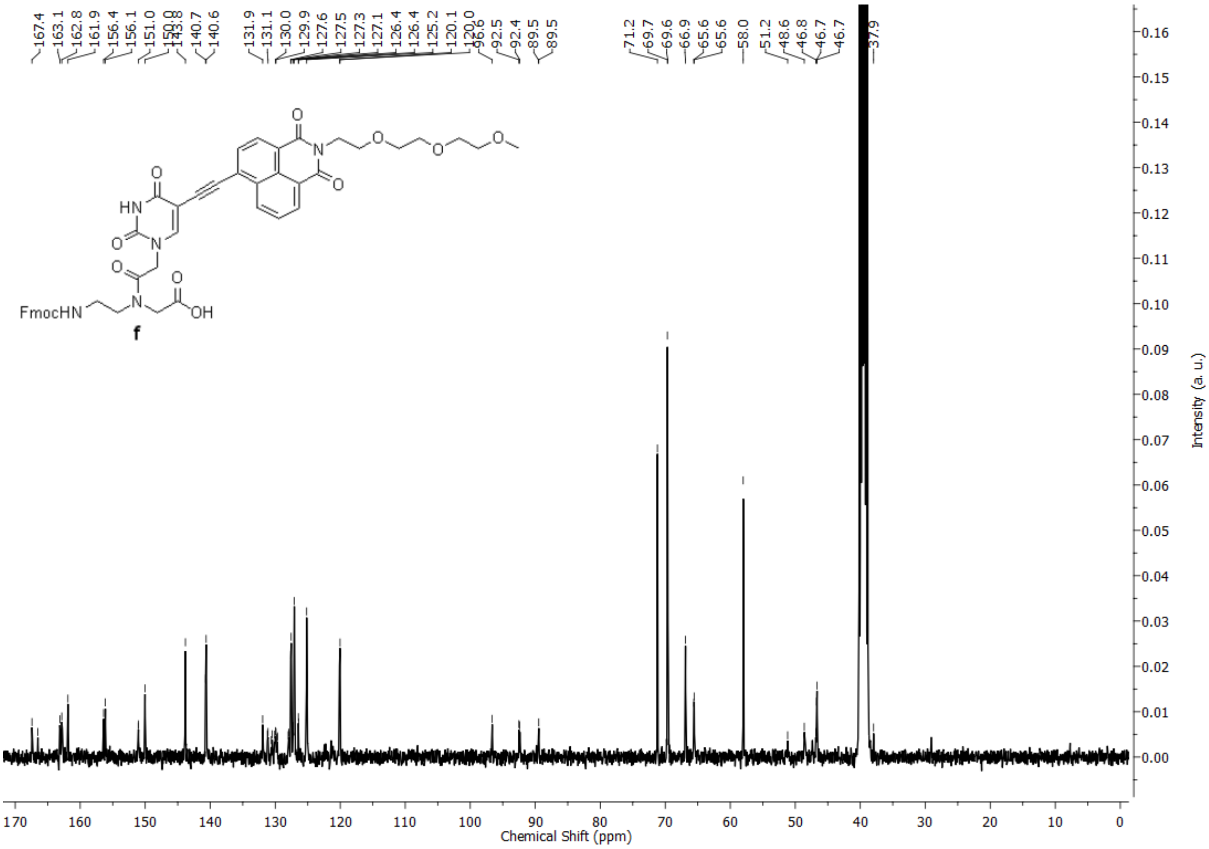

11. Reference

[S1] P. M. Sabale, J. T. George, S. G. Srivatsan, *Nanoscale* **2014**, *6*, 10460–10469.

[S2] A. A. Tanpure, S. G. Srivatsan, *ChemBioChem* **2014**, *15*, 1309–1316.

[S3] E. Beall, S. Ulku, C. Liu, E. Wierzbinski, Y. Zhang, Y. Bae, P. Zhang, C. Achim, D. N. Beratan, D. H. Waldeck, *J. Am. Chem. Soc.* **2017**, *139*, 6726−6735

[S4] S. Fery-Forgues, D. Lavabre, *J. Chem. Educ.* **1999**, *76*, 1260–1264.

[S5] G. Jones II, W. R. Jackson, C.-Y. Choi, *J. Phys. Chem.* **1985**, *89*, 294–300.

[S6] P. M. Sabale, S. G. Srivatsan, *ChemBioChem* **2016**, *17*, 1665–1673.

[S7] P. E. Nielsen, M. Egholm, Peptide nucleic acids-protocols and applications. Horizon Scientific Press, Wymondham, **1999**.

[S8] a) S. Shandrick, Q. Zhao, Q. Han, B. K. Ayida, M. Takahashi, G. C. Winters, K. B. Simonsen, D. Vourloumis, T. Hermann, *Angew. Chem. Int. Ed.* **2004**, *43,* 3177–3182; b) V. K. Tam, D. Kwong, Y. Tor, *J. Am. Chem. Soc.* **2007**, *129*, 3257–3266; c) A. A. Tanpure, S. G. Srivatsan, *Nucleic Acids Res.* **2015**, *43*, e149.
